# Differential regulation of the anthocyanin profile in purple kiwifruit (*Actinidia* species)

**DOI:** 10.1038/s41438-018-0076-4

**Published:** 2019-01-01

**Authors:** Yongyan Peng, Kui Lin-Wang, Janine M. Cooney, Tianchi Wang, Richard V. Espley, Andrew C. Allan

**Affiliations:** 1 School of Biological Sciences, University of Auckland, Auckland, New Zealand; 2 The New Zealand Institute for Plant and Food Research Limited, Mt Albert, Auckland, New Zealand; 3 The New Zealand Institute for Plant and Food Research Limited, East Street, 3214 Hamilton, New Zealand

**Keywords:** Secondary metabolism, Plant biotechnology, Plant breeding

## Abstract

Anthocyanins are a group of secondary metabolites that colour fruit and flowers orange, red, purple or blue depending on a number of factors, such as the basic structure, co-pigmentation, metal ion complexation and vacuolar pH. The biosynthesis of anthocyanin is regulated at the transcriptional level by a group of transcription factors, the MYB–bHLH–WD40 (MBW) complex. In this study, the purple colouration in several kiwifruit (*Actinidia)* species was identified and characterised as red cyanidin-based and blue delphinidin-based anthocyanins. The differential pigmentation in the skin and flesh can be attributed to the differential ratio of cyanidin and delphinidin derivatives accumulated in the total anthocyanin profile. The expression of anthocyanin biosynthetic genes chalcone synthase (*CHS*), flavonoid 3-O-glucosyltransferase (*F3GT*), flavonoid 3′-hydroxylase (*F3*′*H*) and flavonoid 3′5′-hydroxylase (*F3*′*5*′*H*) is crucial for anthocyanin accumulation. However, the balance of expression of the *F3*′*H* and *F3*′*5*′*H* genes appears responsible for the ratio of cyanidin and delphinidin derivatives, while a lack of *CHS*, *F3GT* and *MYB110* expression is responsible for a lack of total anthocyanins. The transcriptional regulation of the *F3*′*H* and *F3*′*5*′*H* promoters by the R2R3 MYB transcription factor MYB110 is markedly different in tobacco transient assays. When kiwifruit MYB10 or MYB110 are over-expressed in *Actinidia chinensis* both cyanidin-based and delphinidin-based anthocyanins are elevated, but *F3*′*H* and *F3*′*5*′*H* genes are not strongly correlated with MYB expression. These results suggest that the core kiwifruit anthocyanin pathway genes are dependent on characterised MYB transcription factors, while other regulatory proteins are more directly responsible for the expression of the *F3*′*H* and *F3*′*5*′*H* genes.

## Anthocyanins: Coloring Kiwifruit

The genetic processes that determine the colors in purple kiwifruit have been revealed by researchers in New Zealand. Anthocyanin pigments are not only essential for plant growth and development, but also provide color to make fruit attractive to pollinators and indicate ripeness for the food industry. Andrew Allan and co-workers at the University of Auckland and The New Zealand Institute for Plant and Food Research Limited examined several different kiwifruit species, and found that their color was determined by the ratio of red cyanidin-based to blue delphinidin-based anthocyanins. The team identified four biosynthetic genes that are crucial for accumulating anthocyanins, and showed that two of those genes control the red-to-blue pigment ratio. This new understanding of the anthocyanin profile could allow metabolic engineering of novel fruit colors, possibly with greater health benefits for humans.

## Introduction

Anthocyanins are flavonoids that belong to the polyphenols group. These secondary metabolites are synthesised in plants and are essential for plant growth and development^1^. Anthocyanin is well recognised for colouring fruits and flowers in a range of hues from orange, red, to blue and violet, making them attractive to pollinators and dispersers, while also serving as an indicator of fruit quality and ripeness for consumers^1,2^. In addition to colour pigmentation in plants, anthocyanin accumulation in leaves of plants and skin of fruits may also absorb high light energy and scavenge free radicals to offer protection against photo-oxidative effects^3^. Furthermore, there have been many studies showing the protective effect of anthocyanin against oxidative stress, inflammation, certain cancers and age-related diseases^4–7^.

Anthocyanin is synthesised from a branched phenylpropanoid pathway by a cascade of biosynthetic enzymes and is regulated at the transcriptional level. While co-pigmentation, different pH level and metal ion complex formations can moderately change the visual colours of anthocyanins, the biosynthetic genes flavonoid 3′ hydroxylase (*F3*′*H*) and flavonoid 3′, 5′-hydroxylase (*F3*′*5*′*H*) are responsible for the type of anthocyanin accumulated^8^. *F3*′*H* belongs to the CYP75B subfamily of the cytochrome P450-dependent monooxygenase (P450) superfamily and converts dihydrokaempferol to dihydroquercetin which is the precursor of cyanidin-based anthocyanin. *F3*′*5*′*H* belongs to the CYP75A subfamily of the P450 superfamily and converts dihydrokaempferol and dihydroquercetin to dihydromyricetin, which is the precursor of delphinidin-based anthocyanin^9^. It appears that the F3′5′H enzyme has evolved from F3′H precursors several times in flowering plants^10^.

Studies in a broad range of plant species have shown that the overall anthocyanin accumulation is regulated at the transcriptional level by R2R3 MYB transcription factors (TF), such as *PAP1* (AtMYB75) in *Arabidopsis*, *MYB1* and *MYB10* in apple, *AN2* in *Petunia*, *VvMYB1A* in grape and *FaMYB10* in strawberry^11–16^. These MYBs often partner with a bHLH TF to form a MBW complex involved in the induction of anthocyanin production and are characterised by the conserved R2R3 DNA-binding domains as well as the conserved [A/S/G] NDV motif for anthocyanin-promoting MYB TFs in dicots, the C-terminus KPRPR[S/T]F motif and the bHLH-interacting motif^16–18^. Over-expression of PAP1 in *Arabidopsis* resulted in elevated transcript level of bHLH TF TT8 and *Petunia* bHLH TF AN1 interacts with MYB TF AN2 to regulate anthocyanin pathway^19,20^. In the model plant *Nicotiana tabacum*, transient over-expression of kiwifruit *AcMYB110* revealed that the endogenous bHLH TF *NtAN1* is an activator of anthocyanin biosynthetic genes and is regulated by the other endogenous bHLH TF *Nt*JAF13^21^.

Kiwifruit belongs to the genus *Actinidia* which comprises more than 70 species, with fruit that show different colours and texture in both skin and flesh^22^. The pigment in the red-fleshed kiwifruit (*Actinidia chinensis*) has been previously identified as cyanidin-based anthocyanin and the anthocyanin biosynthetic gene flavonoid 3-O-glucosyltransferase *F3GT1* was responsible for accumulation of anthocyanin^23^. An R2R3 MYB TF *MYB110* was identified from *Actinidia* hybrid population that determines red petal colour and activates *F3GT1* to produce anthocyanin^24^. In terms of fruit colour, *AcMYB75* and *AcMYBF110* have been identified from *A. chinensis* for their activation of anthocyanin biosynthetic genes^25,26^. Delphinidin was first reported in two taxa: *A. melanandra* and *A. arguta* var. *purpurea* but the genes regulating its accumulation remain to be studied^27^. Recently it has been reported that small amounts of delphinidin derivatives are present in the red-inner pericarp of *A. chinensis* ‘Hongyang’^26^.

The final ratio of 3′-hydroxylated derivatives to 3′5′-hydroxylated anthocyanins has been a focus of attempts to engineer flower colour, such as the blue rose, which required both introduction of transgenes for F3′5′H and DFR as well as suppression of the endogenous DFR^28^. In chrysanthemum, over-expression of a daisy F3′5′H and suppression of the endogenous F3′H did not result in more delphinidin derivatives^29^, while a pansy F3′5′H under the regulation of a CHS promoter resulted in delphinidin production in chrysanthemum flower petals^30^. Differential regulation of these genes at the transcriptional level has been studied. In grape, genes encoding F3′5′H and F3′H are expressed in tissues accumulating flavonoids, especially in skin of ripening red berries; there appear to be at least 15 paralogues of the F3′5′H and two *F3′H* genes^31^. The transcript profiles during véraison (colour change) correlate with accumulation of cyanidin-based and delphinidin-based anthocyanins^32^. Climatic effects have been studied in grape, where the ratio of F3′5′H to F3′H may be lowered due to hot temperatures close to harvest^33^. UVB levels appear to differentially affect expression of *F3′H/F3′5′H* genes in Antarctic moss, *Pohlia nutans*^34^, while in grape, light exclusion increases the ratio of dihydroxylated to trihydroxylated anthocyanins and the ratio of expression of *F3′H/F3′5′H*^35^.

In grape, two copies of the gene encoding the F3′H enzyme are present in the grape genome; only one is expressed in vegetative and reproductive tissues. A detailed study of this *F3′H* gene showed it to be well expressed in many tissues, but elevated in berry skins^36^. Its promoter was isolated and used to identify transcriptional regulators. Interestingly, VvMYBA1 (the MYB activator of anthocyanin biosynthesis) was not identified as an interactor, but the interactors isolated included grape MYB repressors, NACs, EIN3 and homeodomain TFs^36^. Furthermore, the grape MYBA1 was responsible for *F3*′*5*′*H* activation and induced the production of trihydroxylated anthocyanins whereas the two closely related MYB TFs, MYBA6 and MYBA7, were unable to activate the trihydroxylated branch of the anthocyanin pathway^37^. In kiwifruit, a *F3′5′H* gene has been isolated and shown to generate delphinidin-based anthocyanins when transiently expressed in tobacco and kiwifruit^38^. Furthermore, insertion of more MYB-binding sites into the promoter of this F3′5′H upregulates delphinidin-derivative content when transiently over-expressed in the presence of kiwifruit MYB110^38^. Here, we aim to characterise the key biosynthetic genes that are necessary for accumulation of anthocyanin in kiwifruit, and to ascertain if there is differential regulation of *F3′H* and *F3′5′H* that contributes to the ratio of cyanidin and delphinidin in different kiwifruit species.

## Results

### Colour development during ripening

**Fig. 1:**
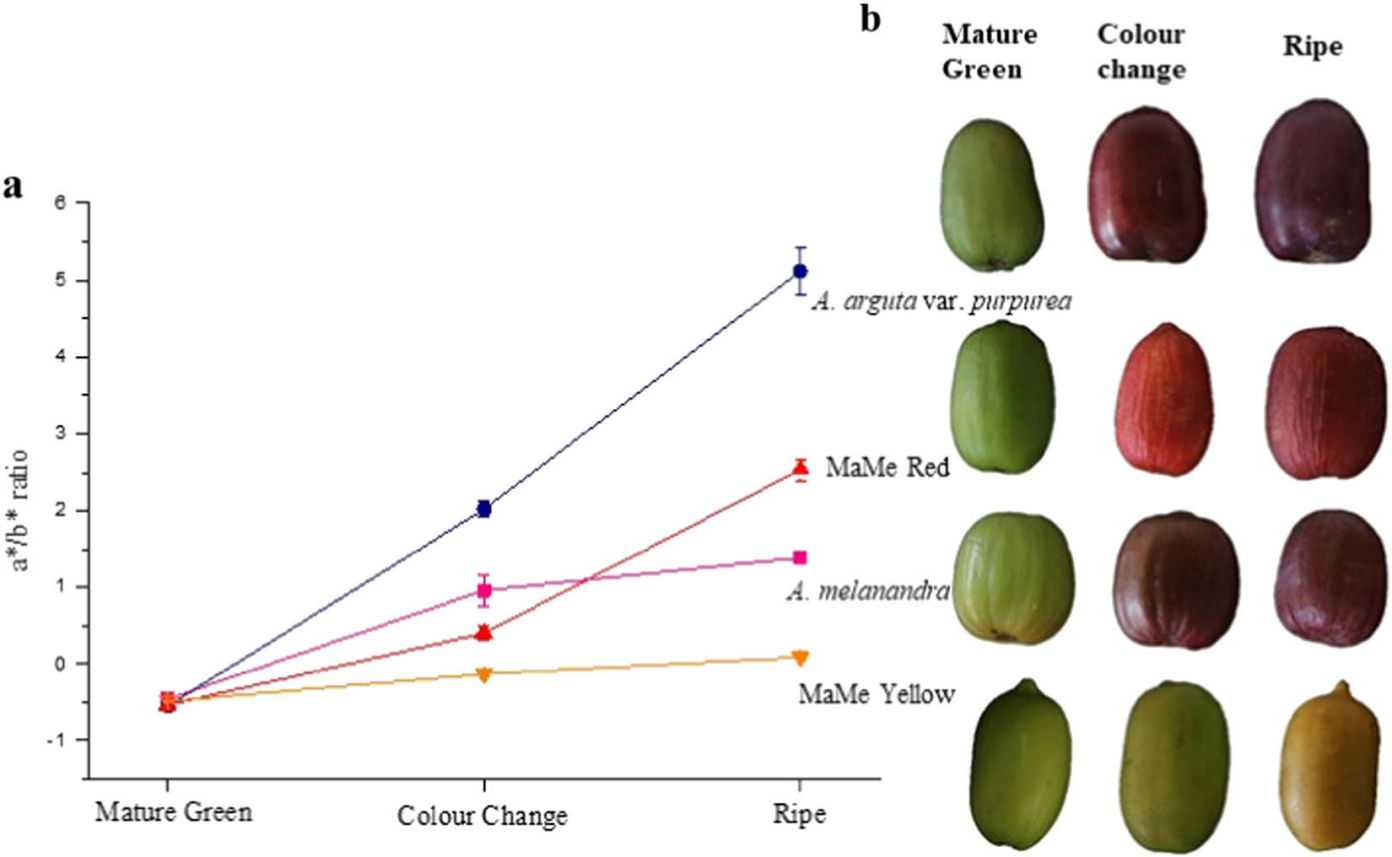
Colour change of *Actinidia melanandra, A. arguta* var. *purpurea, A. macrosperma* × *A. melanandra* (MaMe) Red and MaMe Yellow. **a** Chromameter measurement shown as a*/b* ratio where a* represents green–red axis and b* represents yellow–blue axis. High a*/b* ratio indicates change of colour from green and yellow towards red and blue. Error bars are the SEM for four biological replicates. **b** Digital images showing the colour development from mature green stage to ripe stage

The fruits were harvested at the mature green stage as defined by 100% black seed coat colour, average firmness between 636 and 980 kgf, average dry matter of 10% and average degrees Brix of 4. The fruits were then ripened at 20 °C. Hue angles as measured by Minolta CR300 showed that all four *Actinidia* species had similar green/yellow skin colour at harvest (Fig. 1a, b). As the fruit ripened, the intensity of red hues increased on the skin and eventually blue hues developed, as detected by Minolta chromameter. The deepest colour was in the skin of *A. arguta* var. *purpurea* (Ap), followed by *A. macrosperma* × *A. melanandra* (MaMe) Red and *A. melanandra* (Me). Only the MaMe Yellow remained at low a*/b* ratio which indicates yellow hues. A similar colour development was observed in the fruit flesh, changing from green to deep red at the ripe stage (Supplemental Fig. 1). The exception was the MaMe Yellow kiwifruit where both the skin and flesh colour developed from green to yellow at the colour-change stage, and then to orange at the ripe stage.

### Detection of cyanidin and delphinidin in fruit skin and flesh

Anthocyanins were analysed by HPLC and LCMS in the skin and flesh of the ripening fruit lines. There was no detected anthocyanin at the mature green fruit stage. At both colour-change stage and ripe stage, anthocyanin accumulation was higher in the skin than in the flesh, hence the intense pigmentation observed in the skin. Cyanidin-3-*O*-*β*-galactoside (cy-gal), cyanidin 3-*O*-[2-*O*-(*β*-xylosyl)-*β*-galactoside] (cy-xylgal) and delphinidin 3-*O*-[2-*O*-(*β*-xylosyl)-*β*-galactoside] (dp-xylgal) were detected in the purple kiwifruit (Supplemental Fig. 3) as previously reported^27,39^. The most abundant cyanidin-based compound was cy-xylgal, comprising between 20 and 99% of total anthocyanins.

**Fig. 2:**
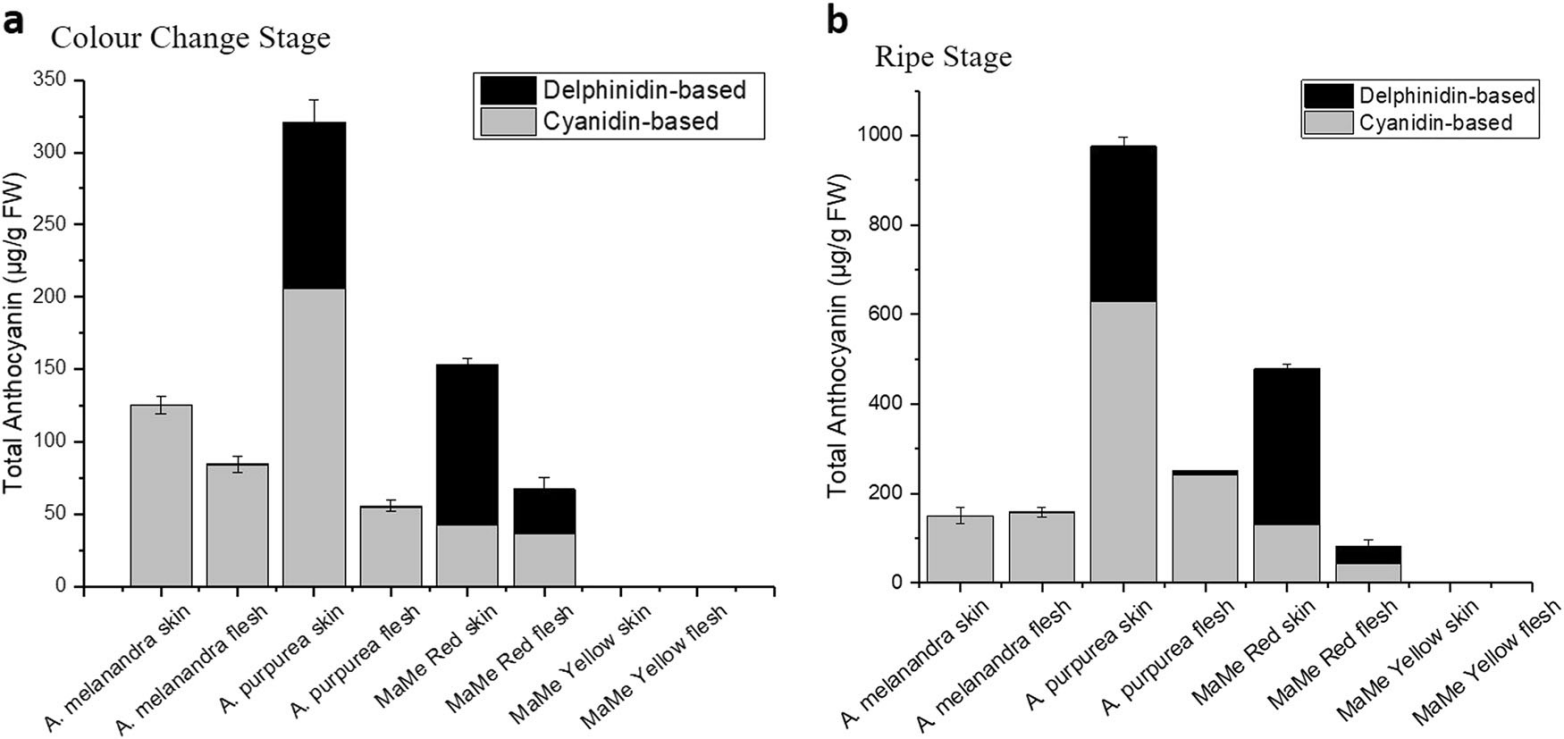
Anthocyanin content in *Actinidia* species. Quantification of cyanidin-based and delphinidin-based anthocyanins in the skin and flesh of *Actinidia melanandra, A. arguta* var. *purpurea*, MaMe Red and MaMe Yellow during colour-change stage (**a**) and ripe stage (**b**). Anthocyanins were measured as cyanidin-3-galactoside equivalents. Error bars are the SEM for three biological replicates

The two cyanidin-based anthocyanins comprised over half of the total anthocyanin profile in the skin and flesh tissue of Me and Ap, while the delphinidin-based anthocyanin was predominant in the skin and flesh tissue of MaMe Red (Fig. 2). Delphinidin was detected in Me skin in relatively small amounts. The highest total anthocyanin accumulation at ripe stage was measured for Ap, which accumulated 976 μg/g FW in skin tissue and 250 μg/g in flesh, followed by MaMe Red (478 μg/g in skin, 81 μg/g in flesh) and Me (150 μg/g in skin, 159 μg/g in flesh). No anthocyanin was detected in MaMe Yellow kiwifruit. At the ripe stage, MaMe Red skin comprised 73% delphinidin-based and 27% cyanidin-based anthocyanin (Supplemental Table 1). The proportion of delphinidin derivatives and cyanidin derivatives in the flesh was 46 and 54%, respectively. In Ap, cyanidin-based anthocyanins contributed to 64% of the total anthocyanin in the skin and 97% in the flesh. In contrast, cyanidin-based anthocyanins contributed to almost all the measured anthocyanin in skin and flesh in Me.

It appears that the total anthocyanin level increased as the fruit transitioned from colour-change stage to ripe stage but the proportion of cyanidin and delphinidin remained at a similar ratio (Fig. 2).

### Expression analysis of anthocyanin biosynthetic genes during fruit colour change

Two key enzymes responsible for the synthesis of cyanidin-based and delphinidin-based anthocyanins are the closely related flavonoid 3′-hydroxylase (*F3*′*H*) and flavonoid 3′, 5′-hydroxylase (*F3*′*5*′*H*)^9^. In the recently published genome of *Actinidia chinensis*^40^, there are three *F3*′*H*-like and two *F3*′*5*′*H*-like genes. Previously, gene expression of two of the *AcF3*′*H* genes was detected in the red-fleshed *A. chinensis*, which contains cyanidin 3-*O*-[2-*O*-(*β*-xylosyl)-*β*-galactoside]^23^.

Recently, the *AcF3*′*5*′*H* gene has been shown to produce delphinidin-based anthocyanin in *Agrobacterium*-mediated transient assays in tobacco and kiwifruit^38^. The DNA sequence of *F3*′*H* and *F3*′*5*′*H* genes was acquired from the reference *A. chinensis* genome and was cloned from all four *Actinidia* species used in this study.

**Fig. 3:**
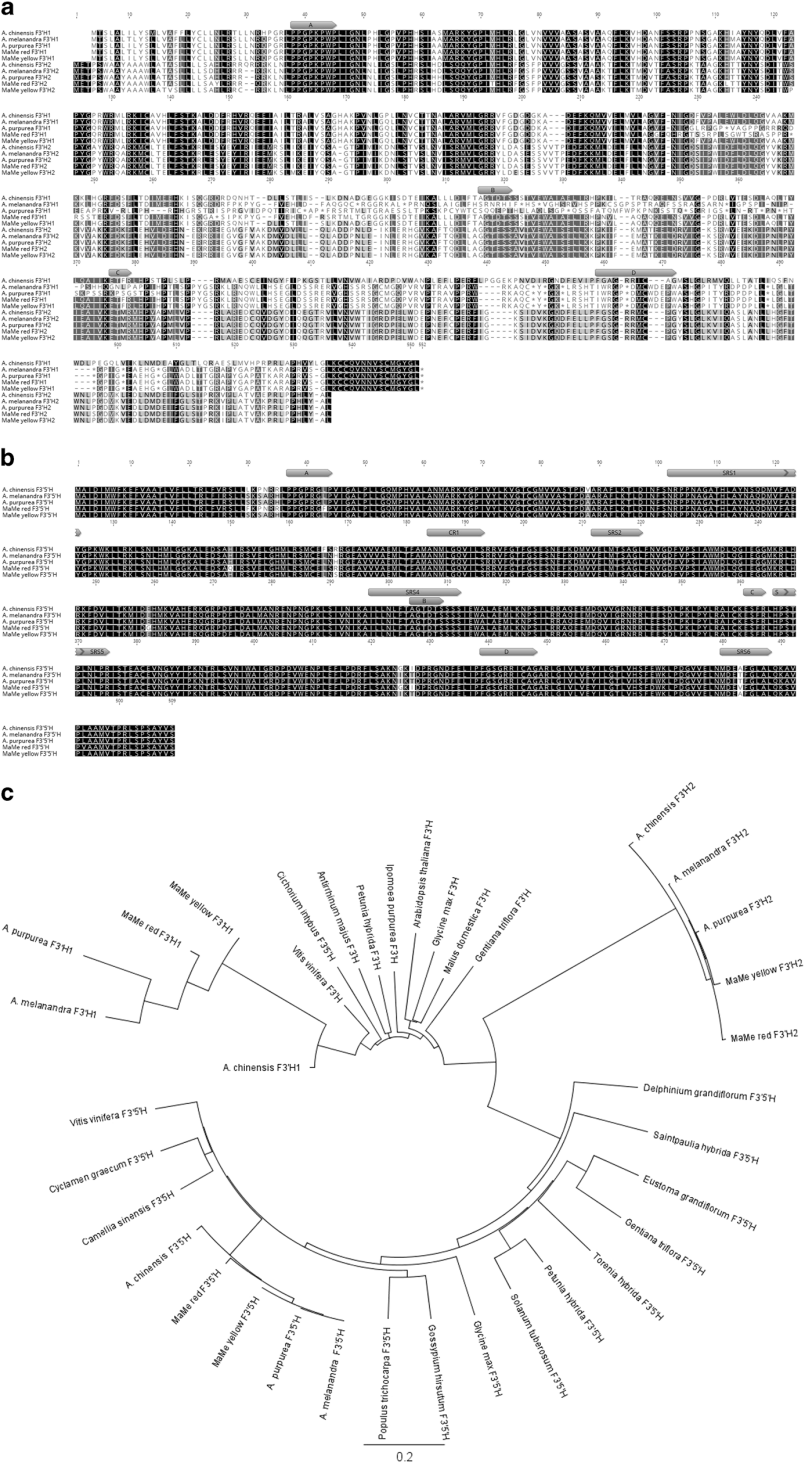
Deduced amino acid sequences of Flavonoid 3′ Hydroxylases and Flavonoid 3′5′ Hydroxylases. **a** Alignment of F3′H1 and F3′H2 from *Actinidia chinensis, A. melanandra, A. arguta* var. *purpurea*, MaMe Red and MaMe Yellow. The deduced amino acid sequences predicted multiple stop codons throughout the F3′H1 sequences except for the *A. chinensis*. **b** Alignment of F3′5′H sequences from the above mentioned species. Motifs specific to P450 enzymes are shown in both alignment A and B. Box A: proline-rich hinge region, box B: oxygen-binding pocket motif, box C: EXXR motif, box D: heme-binding domain. Substrate recognition sites (SRS) 1, 2, 4, 5, 6 and functional domains for hydroxylation activity (CR1) are labelled in F3′5′H amino acid alignment. **c** Phylogenetic relationship between F3′Hs and F3′5′Hs from kiwifruit and other plant species. Deduced amino acid sequences were aligned by global alignment with free end gaps. The protein distance was calculated by Jukes-Cantor model and the tree was constructed by neighbour-joining method using 1000 bootstrap replicates in Geneious 10.0.3

Deduced amino acid alignments have shown several stop codons throughout the F3′H1 from the purple kiwifruit lines, which suggests loss of function of F3′H1 for anthocyanin accumulation (Fig. 3a). F3′H2 appeared to be the functional enzyme and the sequences were well conserved across all *Actinidia* species tested. F3′5′H amino acid sequences share very high similarity among *Actinidia* species and the substrate recognition sites and reported functional domains for hydroxylation activity were highly conserved (Fig. 3b)^31^. The P450 enzyme characteristics were reflected in the kiwifruit F3′Hs and F3′5′Hs by the presence of a proline-rich hinge region, oxygen-binding pocket, EXXR motif and the heme-binding domain^41,42^.

Phylogenetic analysis revealed that the *Actinidia* F3′Hs were closely related to the *A. chinensis* F3′H and clustered with F3′Hs from other plant species (Fig. 3c). The *Actinidia* F3′5′Hs clustered with the *A. chinensis* F3′5′H within the F3′5′H clade from other plant species.

**Fig. 4:**
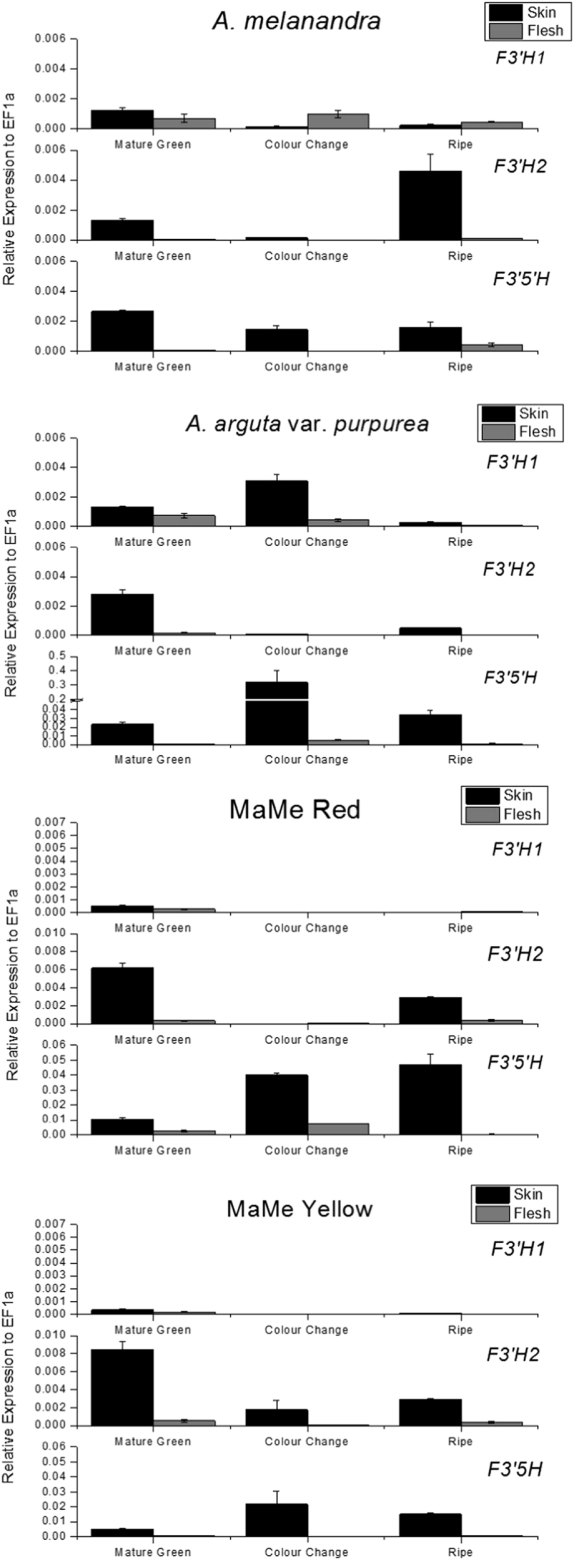
Expression of *F3*′*H1, F3*′*H2* and *F3*′*5*′*H* in *Actinidia melanandra, A. arguta* var. *purpurea*, MaMe Red and MaMe Yellow. Expression was measured in the skin and flesh tissue across the three developmental stages: mature green, colour change and ripe. Error bars are SEM for three biological replicates

Two *F3*′*H*-like genes and one full-length *F3*′*5*′*H*-like gene were expressed in the skin and flesh of purple fruit lines. Transcript of the *F3*′*5*′*H* was detected in the skin at all developmental stages in all kiwifruit species (Fig. 4), but less so in the flesh. Moreover, the expression of the *F3*′*H*s and *F3*′*5*′*H* was detected at the mature green stage when there was no anthocyanin detected. The expression of *F3*′*5*′*H* was the highest in Ap skin at the colour-change stage. MaMe Red skin also showed high levels of *F3*′*5*′*H* expression at the colour-change stage and the ripe stage. *F3*′*5*′*H* expression was lower in Me compared to that in Ap and MaMe Red. Of the two *F3*′*H*-like genes, *F3*′*H2* appeared to have higher expression than *F3*′*H1* in Me, MaMe Red, Yellow and Ap, except for when its low expression at colour-change stage coincided with the slightly higher *F3*′*H1* expression in Ap.

Pearson’s correlation showed that the expression of *F3*′*5*′*H* is correlated with total anthocyanin accumulation (*r* = 0.87, *p* = 0.0024**) at colour-change stage (Table 1), and that the expression of *F3*′*H1* correlated strongly with the accumulation of cyanidin-based anthocyanin (*r* *=* 0.85, *p* = 0.0041**).

**Table 1:** Pearson’s correlation comparing relative gene expression in fruit at colour-change stage with anthocyanin concentration

	**Cyanidin-based anthocyanin**		**Delphinidin-based anthocyanin**		**Total anthocyanin accumulation**	
	Pearson’s coefficient	*p*-Value	Pearson’s coefficient	*p*-Value	Pearson’s coefficient	*p*-Value
*F3*′*H1*	0.8457	0.0041**	0.5449	0.0813	0.8319	0.0052**
*F3*′*H2*	−0.3414	0.7961	−0.2454	0.721	−0.3489	0.8015
*F3*′*5*′*H*	0.7691	0.0128*	0.7326	0.0194*	0.8707	0.0024**
*CHS*	0.8049	0.008**	0.8146	0.0069**	0.9358	<0.001 ***
*CHI*	0.8078	0.0077**	0.6695	0.0347*	0.8651	0.0028**
*F3H*	0.2179	0.3021	0.5681	0.0709	0.422	0.1488
*DFR*	0.6028	0.0569	0.9706	<0.001 ***	0.8764	0.0021**
*LDOX*	−0.2189	0.6987	0.3401	0.2049	0.0204	0.4808
*F3GT*	0.8674	0.0026**	0.7802	0.0112**	0.9601	<0.001 ***

Values showing statistically significant correlation; *p < 0.05; **p < 0.01; ***p < 0.001

Considering the multiple stop codons throughout the deduced amino acid sequence of *F3*′*H1*s, *F3*′*H2* appears to be the functional enzyme converting substrates to cyanidin-based anthocyanins. However, when considering all tissues, *F3*′*H2* has a statistically negative association with total anthocyanin accumulation. This is due to the expression of *F3*′*H2* in the absence of anthocyanin in MaMe Yellow.

The expression of other biosynthetic genes in the anthocyanin pathway was analysed in both skin and flesh tissue across the three developmental stages (Supplemental Figs. 2A and 2B). Gene sequences were mined from the recently published *A. chinensis* genome^40^. Chalcone synthase (*CHS*) and flavonoid 3-O-glucosyltransferase (*F3GT*) were well expressed in both the skin and flesh of purple kiwifruit but barely detected in MaMe Yellow skin and flesh.

Pearson’s correlation shows that the expression of *CHS* and *F3GT* was strongly correlated with the total anthocyanin accumulation (*r* = 0.94, *p* < 0.001*** and *r* = 0.96, *p* < 0.001***, respectively) (Table 1). Furthermore, chalcone isomerase (*CHI*) and dihydroflavonol reductase (*DFR*) were also associated with total anthocyanin accumulation (*r* *=* 0.87, *p* = 0.0028** and *r* *=* 0.88, *p* = 0.0021**, respectively), in which *DFR* shows strong correlation with delphinidin-derivative accumulation (*r* = 0.97, *p* *<* 0.001***).

Flavonone 3-hydroxylase (*F3H*) and leucoanthocyanidin dioxygenase (*LDOX*) were highly expressed in all the kiwifruit (even fruit without anthocyanin). While *DFR* and *CHI* were correlated with anthocyanin accumulation, the lack of expression of *CHS* and *F3GT* in MaMe Yellow suggests these two genes may limit final anthocyanin levels, while the ratio of the expression of *F3*′*H2* and *F3*′*5*′*H* determines the profile of the accumulated anthocyanins.

### Expression analysis of anthocyanin-associated MYB transcription factors

**Fig. 5:**
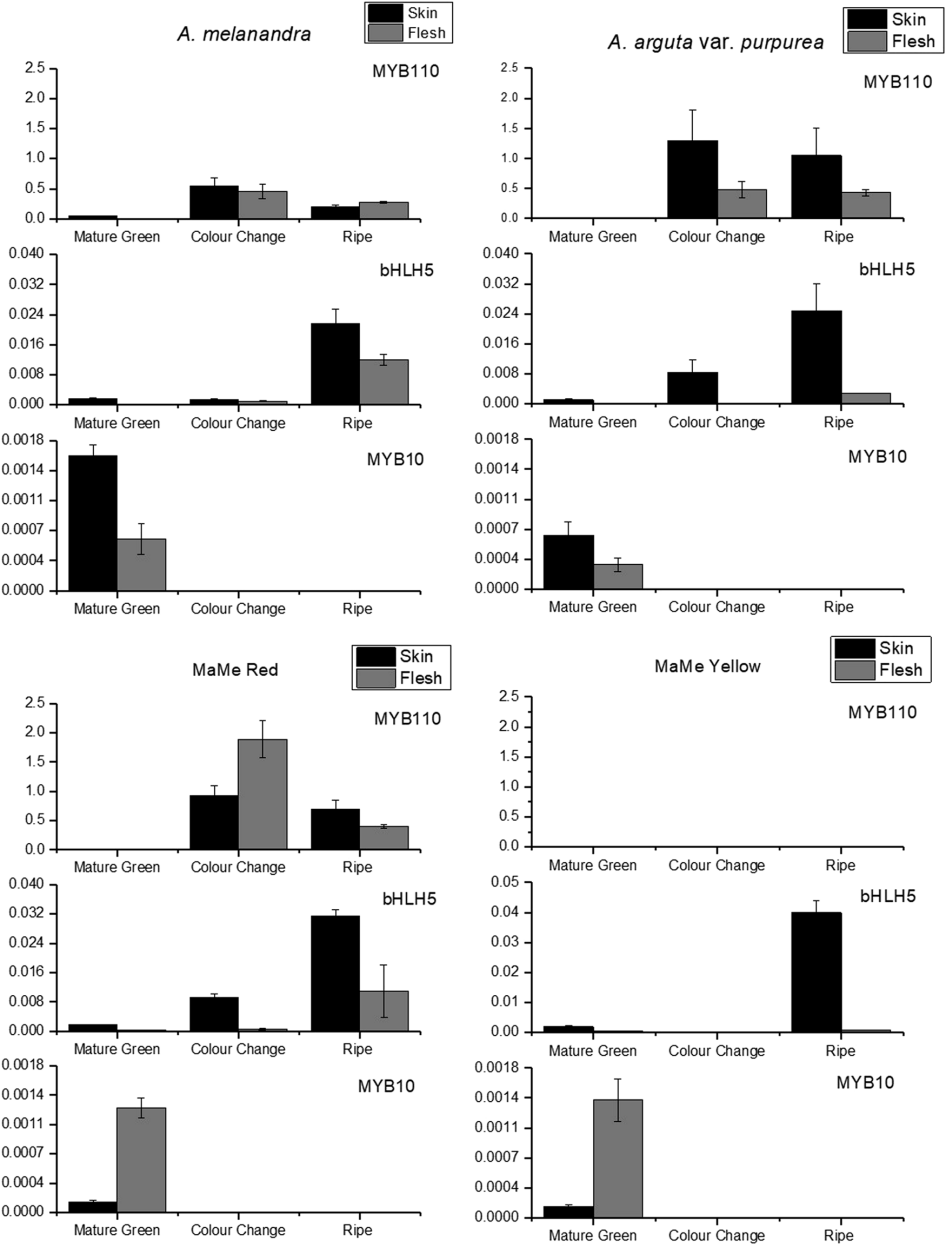
Expression of *MYB110, bHLH5* and *MYB10* in *Actinidia melanandra, A. arguta* var. *purpurea*, MaMe Red and MaMe Yellow. Expression was measured in the skin and flesh tissue across the three developmental stages: mature green, colour change and ripe. Error bars are SEM for three biological replicates

Two R2R3 MYB transcription factors were analysed for their expression associated with anthocyanin accumulation across the three developmental stages. *MYB10* has been implicated in fruit coloration in *A. chinensis*^43^ while *MYB110* controls petal colour in *A. eriantha*^24^. *MYB10* expression was very low at the mature green stage for all kiwifruit and became undetectable in the later stages, suggesting its transcription is not associated with anthocyanin accumulation (Fig. 5). However, *MYB110* was highly expressed in all three purple kiwifruit, particularly the skin, but absent in the MaMe Yellow kiwifruit.

A very strong correlation existed between *MYB110* expression and anthocyanin accumulation at ripe stage (*r* = 0.95, *p* < 0.001***) (Supplemental Table 2). Two potential bHLH TF partners of the anthocyanin-associated MYB were also analysed^21^. The expression of *bHLH2* was undetectable in any of the developmental stages; however, *bHLH5* was expressed in all stages and in all kiwifruit including MaMe Yellow.

Pearson’s correlation shows expression of *bHLH5* is correlated with total anthocyanin accumulation at colour-change stage (*r* *=* 0.82, *p* = 0.0065**) but not at ripe stage (*r* *=* 0.26, *p* = 0.27) (Supplemental Table 2).

### MYB110 is an anthocyanin-related R2R3 MYB transcription factor

**Fig. 6:**
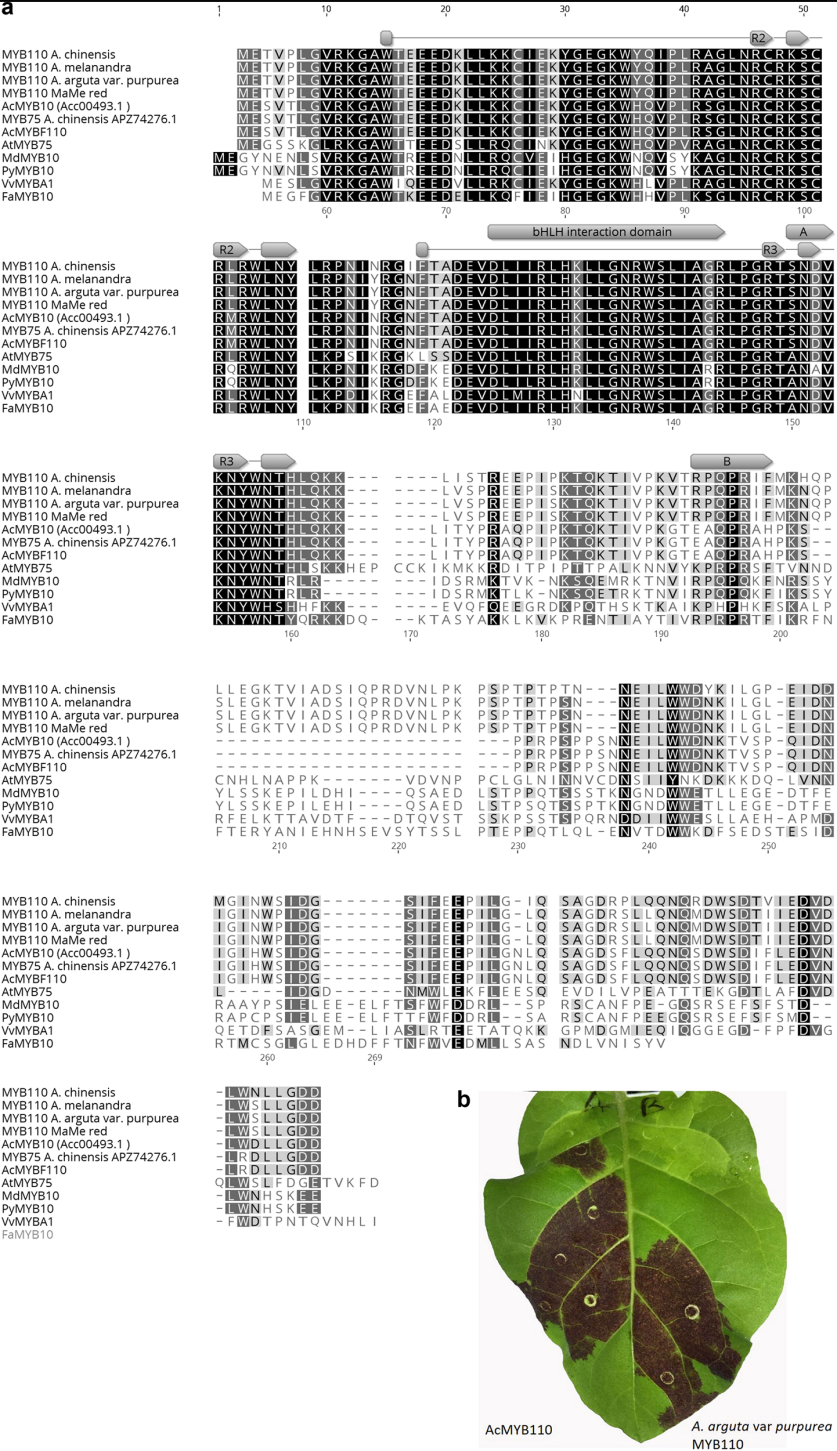
Comparison of kiwifruit MYB sequence and function. **a** Deduced amino acid sequence alignment of AcMYB75, AcMYBF110, MYB10 from *Actinidia chinensis* and other plant species in comparison to MYB110 from *A. chinensis* and the purple kiwifruit lines. The bHLH interaction domain are highlighted in grey box and box A indicates the conserved amino acid residues specific to anthocyanin-promoting MYB in dicot and box B indicates the C terminus motif that is specific to anthocyanin-related regulators. **b** Infiltration site 7 days after transient transformation of *AcMYB110* on the left side of the *N. tabacum* leaf and *MYB110* from *A. arguta* var. *purpurea* on the right side of the leaf

The TF MYB110 was cloned from Me, Ap and MaMe Red kiwifruit for sequence comparison with the known *A. chinensis* MYB110. Alignment showed the protein sequence of Me, Ap and MaMe Red were 100% identical to each other and shared 96% similarity with the AcMYB110 (Fig. 6a).

Alignment with identified anthocyanin-related R2R3 MYB proteins revealed R2R3 DNA-binding domains and the highly conserved bHLH co-factor interacting signature motif ([D/E]Lx2[R/K]x3Lx6Lx3R) in the R2R3 domain of MYB110^17^. Also the conserved [A/S/G]NDV motif for anthocyanin-promoting MYB TFs in dicot and the C-terminus KPRPR[S/T]F motif present in anthocyanin-related regulators was present in the kiwifruit MYB110^18^. Furthermore, infiltration of MYB110 (the identical sequence from either Me, Ap and MaMe Red) induced anthocyanin production in *Nicotiana tabacum* leaves (Fig. 6b), producing 240 ± 41 μg/g FW of delphinidin-based anthocyanin and 434 ± 82 μg/g FW of cyanidin-based anthocyanin (Supplemental Fig. 4). This supports the hypothesis that MYB110 from these species is an anthocyanin-related R2R3 MYB TF and is similar to AcMYB110^21^.

### Transcriptional regulation of *F3*′*H and F3*′*5*′*H* by *MYB110*

*MYB110* was examined for its ability to trans-activate the *Actinidia F3*′*H* and *F3*′*5*′*H* promoters in dual luciferase transient assays in *N. benthamiana* leaves. *AcMYB110* was included in the transient assay as a positive control as it shows high similarity with *MYB110* from purple kiwifruit and has shown strong activation on anthocyanin biosynthetic gene *F3GT* in kiwifruit flower petals^24^. The reporter gene *β-Glucuronidase (GUS)* driven by the 35S promoter was the negative control for background promoter activity.

**Fig. 7:**
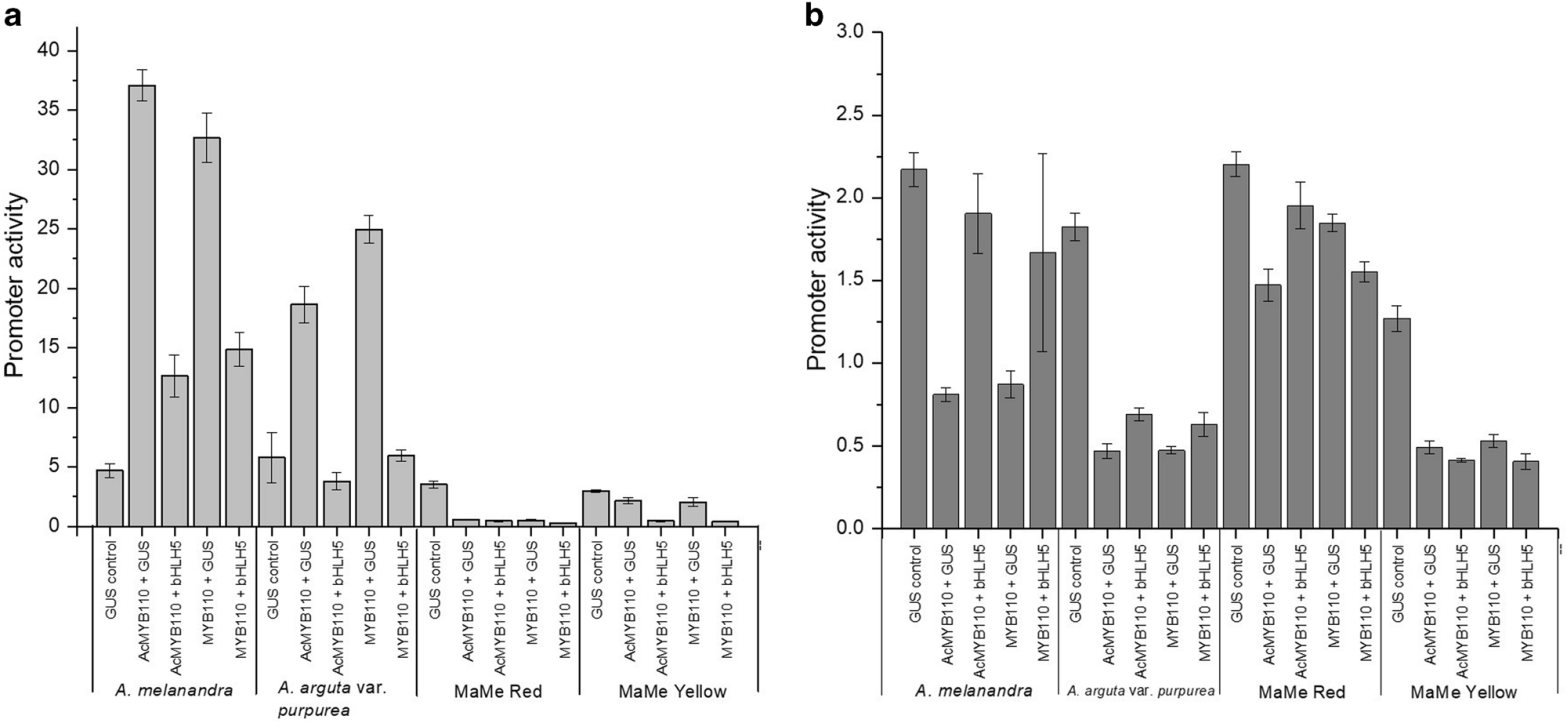
Promoter activation by AcMYB110 and MYB110 cloned from purple kiwifruit lines expressed as LUC/REN activity ratio in dual luciferase transient assay. **a** F3′H promoters isolated from *Actinidia melanandra*, *A. arguta var. purpurea*, MaMe Red and MaMe Yellow are tested for activation by AcMYB110 and MYB110 with and without the transcription factor bHLH5. Error bar indicate SEM for four replicates. **b** F3′5′H promoters isolated from *A. melanandra*, *A. arguta var. purpurea*, MaMe Red and MaMe Yellow are tested for activation by AcMYB110 and MYB110 with and without the transcription factor bHLH5. Error bar indicate SEM for four replicates

The results showed that MYB110 strongly activated certain *F3*′*H* promoters, 33-fold higher in Me and 25-fold higher in Ap (Fig. 7a). This was species specific; there was no activation of the two MaMe *F3*′*H* promoters by either AcMYB110 or MYB110, as their promoter activity readings were lower than the *GUS* control. The same applied for the other published kiwifruit MYB AcMYB10 (data not shown). The potential partner of the MYB, bHLH5, gave no additional activation, possibly due to the strong endogenous activity of the tobacco bHLH, NtAN1^21^.

There was no activation of any of the cloned Actinidia *F3*′*5*′*H* promoters by infiltration with *AcMYB110* or *MYB110*, even in the presence of *bHLH5* (Fig. 7b). Surprisingly each of the promoters showed high background activity (twice as much luciferase produced as 35s-*renillia* without additional transcription factors) and this activity could not be further enhanced by cloned MYBs or bHLHs. Therefore, MYB110 showed strong activation of the Actinidia *F3*′*H* promoters cloned from Me and Ap but seemed unable to activate MaMe *F3*′*H* promoters or any of the *F3*′*5*′*H* promoters. This suggests the *F3*′*H* and *F3*′*5*′*H* promoters are differentially regulated; in some instances the promoter of the *F3*′*H* can be upregulated by MYB110, while the *F3*′*5*′*H* is not directly affected by known kiwifruit MYBs.

### The activity of the F3′H and F3′5′H in *A. chinensis* plants expressing *35s:MYB110* or *35s:MYB10*

**Fig. 8:**
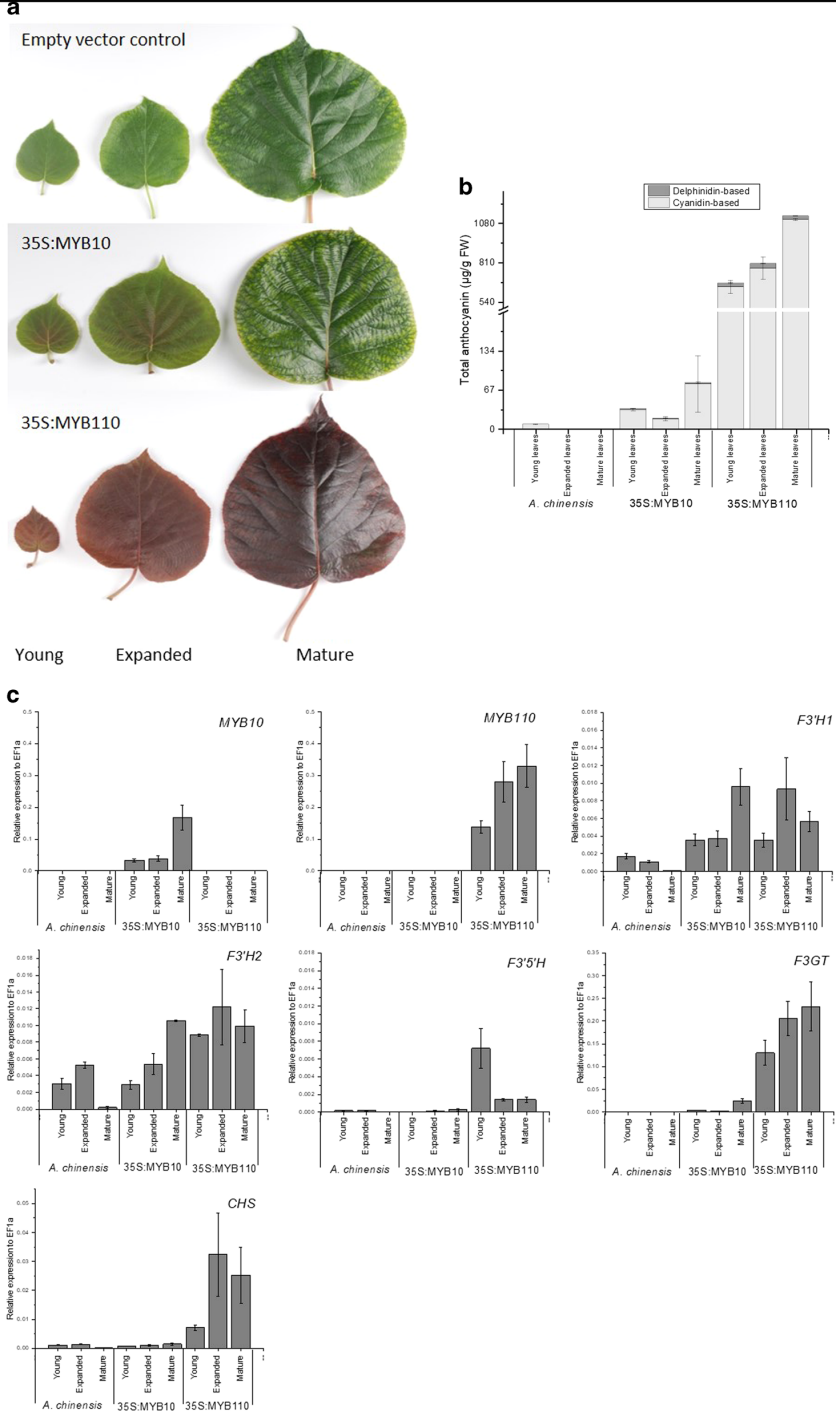
The activity of *F3*′*H* and *F3*′*5*′*H* in *Actinidia chinensis* over-expressing empty 35s vector, 35s:AcMYB10 and 35s:AcMYB110. **a** Digital image of young, expanded and mature leaves from the transformed *A. chinensis* lines. **b** Measurement of cyanidin-based and delphinidin-based anthocyanin in the leaves. Error bars indicate SEM for three independent plant lines. **c** Expression of *MYB10, MYB110* and anthocyanin biosynthetic genes. *F3*′*H* flavonoid 3′ hydroxylase, *F3*′*5*′*H* flavonoid 3′ 5′ hydroxylase*, CHS* chalcone synthase*, F3GT* flavonoid 3-O-glucosyltransferase. Error bars indicate SEM for three independent plant lines

To further examine the regulation of kiwifruit delphinidin biosynthesis, three independent lines of *A. chinensis* over-expressing either *35s:AcMYB10* or *35s:AcMYB110* were examined. Unlike control empty vector plants in the same greenhouse conditions, the leaves of *35s:AcMYB110* were completely red at all stages, while the young and expanded leaves of *35s:AcMYB10* showed red colour in the centre (Fig. 8a).

A small amount of cyanidin derivative (8 μg/g FW) was detected in the young leaves of the empty vector control lines, but not in expanded or mature leaves. In *AcMYB10* over-expression lines, anthocyanin was visible in the centre of the young (33 μg/g FW) and expanded leaves (17 μg/g FW), and was detected along the veins of the mature leaves but this was visibly masked by green colouration (76 μg/g FW) (Supplemental Table 3).

Anthocyanin production was significantly higher in the *AcMYB110* over-expression lines, accumulating 643 μg/g of cyanidin derivative in young leaves, 771 μg/g in expanded leaves and up to 1105 μg/g FW of cyanidin derivative in the large mature leaves.

Delphinidin-derivative was detected in all leaves from *AcMYB10* and *AcMYB110* over-expression line ranging from 1–2 μg/g FW and 25–32 μg/g FW, respectively. Real time quantitative PCR (RT-PCR) revealed that the expression of *MYB110* correlated with the expression of *CHS* as well as *F3GT* in red leaves, which is consistent with previous findings (Fig. 8c)^23^.

Both copies of *F3*′*H* were expressed regardless of the MYB over-expression and the expression of *F3*′*5*′*H* was elevated in MYB110 over-expression line but not to the same extent as the expression of *CHS* and *F3GT*, hence the low-delphinidin accumulation. This suggests that in *A. chinensis*, the regulatory effect of MYB110 is more prominent on *CHS* and *F3GT* and less direct on *F3*′*H* and *F3*′*5*′*H* for anthocyanin accumulation.

## Discussion

The intense pigmentation on both the skin and flesh of the purple kiwifruit is due to the accumulation of cyanidin-based and delphinidin-based anthocyanin. The presence of both cyanidin and delphinidin has been observed in some *A. melanandra* and *A. arguta* var. *purpurea* genotypes^27^. The data presented here show that the key change during the ripening process of *A. melanandra*, *A. arguta* var. *purpurea* and the two MaMe kiwifruits is the increase in total anthocyanin concentration while the composition and the ratio of cyanidin to delphinidin remained the same between the colour-change stage and the ripe stage.

A comparison of the anthocyanin biosynthetic gene transcription between the purple *A. melanandra, A. arguta* var. *purpurea* and MaMe Red, with MaMe Yellow kiwifruit showed that *CHS* and *F3GT* expression were strongly correlated with the accumulation of total anthocyanin. CHS is responsible for initiating flavonoid biosynthesis^44^ and F3GT has been shown to be responsible for anthocyanin biosynthesis in red-fleshed kiwifruit^23^. The anthocyanin biosynthetic genes *F3H* and *LDOX* have high expression in the purple fruits but there was no statistical evidence that their gene expression correlated with the accumulation of anthocyanin. F3H catalyses the hydroxylation of the substrate naringenin to form dihydrokaempferol (DHK), which is the precursor of anthocyanin as well as the metabolic intermediate for flavonols^45^. In a recent publication, *LDOX* was proposed to have a critical role in anthocyanin biosynthesis in *A. arguta* species^46^. *LDOX* is undoubtedly required for processing substrate flux down the anthocyanin biosynthetic pathway, but was less correlated with anthocyanin accumulation than *CHS, F3GT* and *MYB110*. *F3*′*H* and *F3*′*5*′*H* are important for anthocyanin accumulation as *F3*′*H* catalyses the hydroxylation on the intermediate substrate DHK into dihydromyricetin (DHM) at the 3′ position of the B-ring which forms the precursor of cyanidin while *F3*′*5*′*H* catalyses the hydroxylation on both the 3′ and 5′ position which forms the precursor of delphinidin^9^. The expression of these two genes was present in all kiwifruit tissue tested, even in the MaMe Yellow where no anthocyanin was accumulated. In the previously characterised red-centred *A. chinensis*, the red colour was due to the accumulation of cyanidin-based anthocyanin only and no delphinidin-based anthocyanin was detected^23^. In this cultivar of *A. chinensis*, only *F3*′*H1* and *F3*′*H2* gene expression was detected while no transcription of *F3*′*5*′*H* was observed. This suggests that while *CHS* and *F3GT* are crucial for total anthocyanin accumulation, *F3*′*H* and *F3*′*5*′*H* are required for determining anthocyanin composition.

In this study, the ratio of cyanidin to delphinidin appeared to be determined as soon as the colour develops at the colour-change stage. During colour-change stage, the expression of *F3*′*H1* was absent and the expression of *F3*′*H2* was very low, but it appeared to be complemented by the high *F3*′*5*′*H* expression at the colour-change stage and the ripe stage, hence a high amount of delphinidin was accumulated in the MaMe Red kiwifruit. In *A. arguta* var. *purpurea* the expression of *F3*′*H2* was very low at the colour-change stage but was complemented by the high expression of *F3*′*5*′*H* which may explain the similar level of cyanidin-based and delphinidin-based anthocyanin. In addition, *F3*′*H* and *F3*′*5*′*H* were expressed at the mature green stage of all kiwifruit despite the lack of red colour at that stage. There were multiple stop codons at consistent amino acid positions throughout the deduced amino acid sequence of *F3*′*H1* cloned from purple kiwifruit lines, suggesting that *F3*′*H2* was the most likely candidate for the functional enzyme responsible for cyanidin accumulation.

R2R3 MYB transcription factors also play a role in regulating anthocyanin biosynthesis in kiwifruit. Previously, *AcMYB110* has been shown to determine red petal colour in kiwifruit flowers and activated *F3GT* expression^24^. Here, *MYB110* was highly expressed in both the skin and flesh of the purple kiwifruit lines but absent in the MaMe Yellow kiwifruit. The expression showed correlation with total anthocyanin accumulation and coincided with the colour development, as well as the expression of the biosynthetic genes *CHS* and *F3GT*. Protein alignment showed 96% similarity with *AcMYB110*, while the presence of anthocyanin regulator specific motifs and the ability to induce anthocyanin in tobacco leaves suggests a functionally similar MYB activator of anthocyanin biosynthesis. In promoter activation assays in the model plant *N. benthamiana*, *MYB110* induced activation of the *F3*′*H* promoter isolated from *A. melanandra* and *A. arguta* var. *purpurea*, but did not induce activation of the MaMe *F3*′*H* promoters. Moreover, there was no activation of any of the cloned *F3*′*5*′*H* promoters even in the presence of the MBW complex partner *bHLH5*. The TF *bHLH5* was most highly expressed at the ripe stage in all lines, but as it showed low correlation to anthocyanin accumulation and *MYB110* expression, and the transcript appeared to be independent of *MYB110* expression.

Surprisingly both *F3*′*H* and *F3*′*5*′*H* are well expressed in MaMe Yellow kiwifruit despite the absence of *MYB110* expression, which implies MYB110 it is not the key regulator of *F3*′*H* and *F3*′*5*′*H*. MYB110 was found to activate two of the cloned *F3*′*H* promoters, and no *F3*′*5*′*H* promoters, so it is apparent that these two genes are differentially regulated and with other transcription factors likely to regulate anthocyanin profile. In a recent publication, AcMYB110 was only able to induce significant delphinidin accumulation in *A. eriantha* kiwifruit only when the *F3*′*5*′*H* promoter was engineered with the MYB-binding site R6 motif^38^. Otherwise, the AcMYB110 has very little activation on the native *AcF3*′*5*′*H* promoter. It appeared that when AcMYB110 was transiently expressed in *A. eriantha* kiwifruit, there was significantly more cyanidin-based anthocyanin and little delphinidin-based anthocyanin.

In leaves of stably transformed *A. chinensis* lines, *F3*′*5*′*H* expression was elevated with over-expression of *MYB110*, and hence some delphinidin derivatives were produced (2–4% of total anthocyanin). However, the magnitude of induction was significantly less than those of *CHS* and *F3GT. MYB110* over-expression facilitated significant accumulation of cyanidin as the *F3*′*H*s transcripts were already abundant, even in wild-type tissue. Therefore, from results of both stable transgenics and transient assays MYB110 (or MYB10) does not appear to be the main regulator of *F3*′*H* and *F3*′*5*′*H*. In two recent publications, AcMYB75 and AcMYBF110 were reported to be the key R2R3 TFs for anthocyanin regulation in kiwifruit^25,26^. Protein alignment showed that AcMYB75 and AcMYBF110 are both 99.5% identical to *AcMYB10* examined in this study (Fig. 6a). *MYB10* shows no correlation with anthocyanin biosynthesis in purple kiwifruit lines. In *A. chinensis AcMYB75/MYB10* and *AcMYBF110* are important in controlling flesh colour. MYB110 appears to be the activator TF of anthocyanin biosynthesis in purple kiwifruit lines *A. melanandra, A. arguta* var. *purpurea* and MaMe species. It is crucial for anthocyanin accumulation and may partially determine the composition of cyanidin-based and delphinidin-based anthocyanin. However, while it promotes the accumulation of anthocyanin, other TFs or regulatory mechanisms appear responsible for the accumulation of delphinidin-based compounds.

## Conclusion

The anthocyanin biosynthesis pathway has been extensively studied and key transcriptional regulators have been identified. Previous research on anthocyanin biosynthesis in kiwifruit has focused on total anthocyanin accumulation rather than the specific profile of anthocyanins. While it is important to understand the mechanism that switches on the overall pathway, the regulation involved in determining the type of anthocyanin that accumulates will be useful in metabolic engineering. Further research is now required to extend the understanding of the regulation of the anthocyanin profile to achieve novel colours and maximise health benefits.

## Materials and methods

### Plant material and storage condition

*Actinidia melanandra* (Me), *A. arguta var. purpurea* (Ap) and *A. macrosperma* × *A. melanandra* (MaMe) fruits were grown at the Plant and Food Research Orchard, Motueka, New Zealand. Sixty fruits from each vine were collected at the mature green stage. At mature green stage, harvested fruits showed 100% black seed coat colour, average firmness between 636 and 980 kgf, average dry matter between 10 and 12% and average degrees Brix of 4. Twenty fruits were sampled at each stage. For the colour-change stage and ripening stage, all fruits were stored at 20 °C for ripening. Skin peel and flesh tissue were separated, snap-frozen and collected into three biological replicates, each consisting of three fruit.

### Skin and fruit colour measurement

Skin colour change was measured using a Minolta CR-300 chromameter (Konica Minolta, Mahwah, NJ, USA) by the L*a*b* colour space (CIE, 1976)^47^. Measurements were taken on two flat surfaces of each of the four fruits for each stage and repeated twice.

### Anthocyanin identification and quantification

Skin peel and flesh tissue were ground to powder under liquid nitrogen. Anthocyanin concentrations were measured by ultra-high performance liquid chromatography (UHPLC) and the identity of the anthocyanins detected was confirmed by liquid chromatography/mass spectrometry analysis (LC-MS). Approximately 300 mg of skin peel powder and 500 mg of flesh powder for each biological replicate from Me, Ap and two MaMe were freeze-dried overnight. Anthocyanins were extracted with acidified methanol (100% MeOH + 0.1% HCl, 5× volume per material weight) for 2 h at room temperature. A measure of 1 mL of supernatant was spin-dried followed by re-suspension in 500 μL of 20% methanol. The supernatant was filtered through a syringe filter and diluted with 20% methanol prior to analysis. The system used was a Dionex UltiMate 3000 Series UHPLC (ThermoFisher Scientific, San Jose, CA, USA) with PDA (photodiode array) detection at 520 nm. Compound separation was achieved using an Acclaim PA2 C18 column, 3 μ 2.1 × 150 mm (Dionex, ThermoFisher Scientific), maintained at 35 °C. Solvents were (A) 0.1% formic acid and (B) acetonitrile + 0.1% formic acid and the flow rate was 350 μL/min. The initial mobile phase, 100%A was ramped linearly to 92%A at 5 min, then 85%A at 10 min, 80%A at 20 min and 100%B at 34 min. The total run time per sample was 40 min and the sample injection volume was 5 μL. Standards of cyanidin-3-*O*-*β*-glucoside were used to quantitate anthocyanin concentrations and the results for individual and total anthocyanins are reported as cyanidin-3-*O*-*β*-glucoside equivalents per gram fresh weight (FW). For anthocyanin quantification in *A. chinensis* leaves, 300 mg of freeze-dried leave tissue powder were extracted with 2 mL of acidified methanol and used the same procedure as described above.

Identification of detected anthocyanins was confirmed by LC-MS using an LTQ linear ion trap mass spectrometer fitted with an ESI interface (ThermoFisher Scientific, San Jose, CA, USA) coupled to an Ultimate 3000 UHPLC and PDA detector (Dionex, Sunnyvale, CA, USA). Anthocyanin compound separation was achieved using a Poroshell 120 SB-C18 column, 2.7 μ 2.1 × 150 mm (Agilent, Torrance, CA, USA), maintained at 70 °C. Solvents were (A) 5:3:92 acetonitrile:formic acid:water v/v/v and (B) acetonitrile + 0.1% formic acid and the flow rate was 200 μL/min. The initial mobile phase, 100%A was held for 2 min before being ramped linearly to 88%A at 14 min, 5%A at 15 min and held for 4 min before resetting to the original conditions. Sample injection volume was 10 μL. MS data were acquired in the positive mode using a data-dependent LC-MS^3^ method. This method isolates and fragments the most intense parent ion to give MS^2^ data (daughter ions), then isolates and fragments the most intense daughter ion (MS^3^ data). The capillary voltage, capillary temperature, sheath gas pressure and auxiliary gas were set at 43 V, 275 °C, 35 psi and 10 psi, respectively.

### Real time quantitative PCR expression analysis

RNA was isolated from the ground skin and flesh tissue using the Spectrum Plant Total RNA kit (Sigma-Aldrich, www.sigmaaldrich.com) and reverse transcribed into cDNA using the QuantiTect Reverse Transcription kit (Qiagen, www.qiagen.com) according to the manufacturer’s protocol. Genes of the anthocyanin pathway were identified by BLAST with genes of known function on the *Actinidia chinensis* genome and the *Actinidia arguta* RNA-seq data^40,48^ (Supplemental Table 4). Gene-specific primers were designed using Vector NTI 11.0 (http://www.invitrogen.com) and are summarised in supplementary table 5 (Additional file). RT-PCR was carried out using the LightCycler 480 with LightCycler 480 SYBR Green I Master (Roche Diagnostics, USA). Each reaction volume was 5 μL and reactions were run in quadruplicate and a non-template control and water control were included in each run. The thermal cycling conditions were 95 °C for 5 min, followed by 50 cycles of 95 °C for 10 s, 60 °C for 10 s and 72 °C for 20 s, then a melting temperature cycle with continuous fluorescence data acquisition from 65 to 95 °C. The data output was analysed by the lightcycler480 software 1.5 using the Target/Reference ratio to compare the expression level of the target genes normalised to the reference gene, *A. chinensis* orthologue of At1g07940 elongation factor 1-a (EF1α)^49^.

### Isolation, cloning and sequence alignment of candidate genes

Sequences of candidate genes were identified by BLAST from the *A. chinensis* genome^40^ and PCR amplified from cDNA by specific primers (Supplemental Table 5). PCR product of *MYB110* were cloned into pSAK277 over-expression vector using In-Fusion cloning (Takara Bio USA, Inc). *F3*′*H* and *F3*′*5*′*H* genes were amplified using specific primers from the four *Actinidia* species cDNA and cloned in pHEX2 vectors using Gateway cloning (ThermoFisher Scientific, USA). Genes were sequenced by Macrogen, Korea. Multiple nucleotide and amino acid sequence alignment and phylogeny tree were created by Geneious 10.0.3.

### Transient over-expression of MYB110 in tobacco leaves

The vector pSAK277 with 35s:MYB110 construct was transformed into *Agrobacterium tumefaciens* strain GV3101 by electroporation followed by incubation on plate before infiltration. *N. tabacum* plants were grown under glasshouse conditions using natural light with daylight extension to 16 h as described by Espley et al.^12^. Three leaves of 6-week-old *N. tabacum* were used for infiltration and kept under the same growth conditions. Leaves were photographed and harvested 7 days after infiltration in liquid nitrogen and stored at −80 °C until analysis.

### Dual luciferase transient assay in tobacco leaves

Genomic DNA was extract from skin tissue of *A. melanandra*, *A. arguta* var. *purpurea* and MaMe using the DNeasy Plant Mini kit (Qiagen, http://www.qiagen.com/). The 2.3 kb promoter region of *F3*′*H* and *F3*′*5*′*H* were isolated by polymerase chain reaction (PCR), cloned into pGreen 0800-LUC vector using In-Fusion HD cloning (Clontech, Takara Bio USA) and transformed into *A. tumefaciens* GV3101^50^. The promoter sequence was confirmed by sequencing (Macrogen, Korea). Promoter activation dual luciferase assays were performed on leaves of 6-week-old *N. benthamiana* grown under above mentioned glasshouse condition. The promoters of *F3*′*H* and *F3*′*5*′*H* were co-infiltrated with *MYB110*^24^. Three days after inoculation, four leave discs from each treatment were sampled to assay the firefly luciferase and *renilla* luciferase activities using the dual luciferase assay reagent (Promega, Madison, WI, USA). The promoter activities were expressed as a ratio of LUC to REN activity^24,50^.

### Stable over-expression of MYB10 and MYB110 in *A. chinensis*

Newly initiated leaves of *A. chinensis* “Hort16A” from in vitro grown shoots were inoculated with *A. tumefaciens* strain EHA105 containing the vectors of pSAK277 with 35s:MYB10 and 35s:MYB110, respectively. The transformation procedure was based on the previous report^51^. Calli which formed in the regeneration and selection medium containing 150 mg/L of kanamycin were excised individually and transferred to fresh regeneration and selection medium for bud induction. The shoots generated from these independent transformant calli were excised and cultured in the selection medium containing 50 mg/L of kanamycin. After they had rooted, these transgenic plants were potted and grown in the containment greenhouse.

### Statistical analysis

The correlation relationship between the amount of anthocyanin concentration and expression of the anthocyanin biosynthetic genes were calculated and presented in Pearson’s correlation coefficient *r* (Supplemental Table 2).

## Electronic supplementary material

Supplementary Figures and Tables

## References

[1] Glover BJ, Martin C (2012). Anthocyanins. Curr. Biol..

[2] Allan AC, Hellens RP, Laing WA (2008). MYB transcription factors that colour our fruit. Trends Plant. Sci..

[3] Gould KS (2004). Nature’s Swiss army knife: the diverse protective roles of anthocyanins in leaves. Biomed. Res. Int..

[4] Hurst RD (2010). Blueberry fruit polyphenolics suppress oxidative stress‐induced skeletal muscle cell damage in vitro. Mol. Nutr. Food Res..

[5] Li L (2014). Anthocyanin-rich fractions from red raspberries attenuate inflammation in both RAW264. 7 macrophages and a mouse model of colitis. Sci. Rep..

[6] Wu T, Yin J, Zhang G, Long H, Zheng X (2016). Mulberry and cherry anthocyanin consumption prevents oxidative stress and inflammation in diet‐induced obese mice. Mol. Nutr. Food Res..

[7] Peiffer DS (2016). Dietary consumption of black raspberries or their anthocyanin constituents alters innate immune cell trafficking in esophageal cancer. Cancer Immunol. Res..

[8] Zhang Y, Butelli E, Martin C (2014). Engineering anthocyanin biosynthesis in plants. Curr. Opin. Plant. Biol..

[9] Seitz C, Ameres S, Forkmann G (2007). Identification of the molecular basis for the functional difference between flavonoid 3′-hydroxylase and flavonoid 3′, 5′-hydroxylase. FEBS Lett..

[10] Seitz C, Ameres S, Schlangen K, Forkmann G, Halbwirth H (2015). Multiple evolution of flavonoid 3′, 5′-hydroxylase. Planta.

[11] Takos AM (2006). Light-induced expression of a MYB gene regulates anthocyanin biosynthesis in red apples. Plant Physiol..

[12] Espley RV (2007). Red colouration in apple fruit is due to the activity of the MYB transcription factor, MdMYB10. Plant J..

[13] Borevitz JO, Xia Y, Blount J, Dixon RA, Lamb C (2000). Activation tagging identifies a conserved MYB regulator of phenylpropanoid biosynthesis. Plant Cell.

[14] Quattrocchio F (2006). PH4 of petunia is an R2R3 MYB protein that activates vacuolar acidification through interactions with basic-helix-loop-helix transcription factors of the anthocyanin pathway. Plant Cell.

[15] Kobayashi H, Suzuki S, Tanzawa F, Takayanagi T (2009). Low expression of flavonoid 3′, 5′-hydroxylase (F3′, 5′ H) associated with cyanidin-based anthocyanins in grape leaf. Am. J. Enol. Vitic..

[16] Lin-Wang K (2010). An R2R3 MYB transcription factor associated with regulation of the anthocyanin biosynthetic pathway in Rosaceae. BMC Plant Biol..

[17] Zimmermann IM, Heim MA, Weisshaar B, Uhrig JF (2004). Comprehensive identification of *Arabidopsis thaliana* MYB transcription factors interacting with R/B‐like BHLH proteins. Plant J..

[18] Stracke R, Werber M, Weisshaar B (2001). The R2R3-MYB gene family in *Arabidopsis thaliana*. Curr. Opin. Plant Biol..

[19] Tohge T (2005). Functional genomics by integrated analysis of metabolome and transcriptome of Arabidopsis plants over‐expressing an MYB transcription factor. Plant J..

[20] Spelt C, Quattrocchio F, Mol JN, Koes R (2000). Anthocyanin1 of petunia encodes a basic helix-loop-helix protein that directly activates transcription of structural anthocyanin genes. Plant Cell.

[21] Montefiori M (2015). In the Solanaceae, a hierarchy of bHLHs confer distinct target specificity to the anthocyanin regulatory complex. J. Exp. Bot..

[22] Ferguson, A. R. & Huang, H. W. *Horticultural Reviews*. 33 (John Wiley & Sons, Hoboken, NJ, 2007).

[23] Montefiori M (2011). Identification and characterisation of F3GT1 and F3GGT1, two glycosyltransferases responsible for anthocyanin biosynthesis in red‐fleshed kiwifruit (*Actinidia chinensis*). Plant J..

[24] Fraser LG (2013). An R2R3 MYB transcription factor determines red petal colour in an Actinidia (kiwifruit) hybrid population. BMC Genomics.

[25] Li W (2017). Kiwifruit R2R3-MYB transcription factors and contribution of the novel AcMYB75 to red kiwifruit anthocyanin biosynthesis. Sci. Rep..

[26] Liu Y (2017). Expression differences of pigment structural genes and transcription factors explain flesh coloration in three contrasting kiwifruit cultivars. Front. Plant Sci..

[27] Montefiori M, Comeskey DJ, Wohlers M, McGhie TK (2009). Characterization and quantification of anthocyanins in red kiwifruit (Actinidia spp.). J. Agric. Food Chem..

[28] Katsumoto Y (2007). Engineering of the rose flavonoid biosynthetic pathway successfully generated blue-hued flowers accumulating delphinidin. Plant Cell Physiol..

[29] He H, Ke H, Keting H, Qiaoyan X, Silan D (2013). Flower colour modification of chrysanthemum by suppression of f3’h and overexpression of the exogenous senecio cruentus f3'5’h gene. PLoS ONE.

[30] Brugliera F (2013). Violet/blue chrysanthemums—metabolic engineering of the anthocyanin biosynthetic pathway results in novel petal colors. Plant Cell Physiol..

[31] Falginella L (2010). Expansion and subfunctionalisation of flavonoid 3’, 5’-hydroxylases in the grapevine lineage. BMC Genomics.

[32] Castellarin SD (2006). Colour variation in red grapevines (Vitis vinifera L.): genomic organisation, expression of flavonoid 3’-hydroxylase, flavonoid 3’, 5’-hydroxylase genes and related metabolite profiling of red cyanidin-/blue delphinidin-based anthocyanins in berry skin. BMC Genomics.

[33] Li Q (2014). Comparison of distinct transcriptional expression patterns of flavonoid biosynthesis in Cabernet Sauvignon grapes from east and west China. Plant Physiol. Biochem..

[34] Liu S, Ju J, Xia G (2014). Identification of the flavonoid 3′-hydroxylase and flavonoid 3′, 5′-hydroxylase genes from Antarctic moss and their regulation during abiotic stress. Gene.

[35] Guan L (2016). Anthocyanin biosynthesis is differentially regulated by light in the skin and flesh of white-fleshed and teinturier grape berries. Planta.

[36] Sun RZ, Pan QH, Duan CQ, Wang J (2015). Light response and potential interacting proteins of a grape flavonoid 3′-hydroxylase gene promoter. Plant Physiol. Biochem..

[37] Matus JT (2017). A group of grapevine MYBA transcription factors located in chromosome 14 control anthocyanin synthesis in vegetative organs with different specificities compared with the berry color locus. Plant J..

[38] Brendolise C., et al. Multiple copies of a simple MYB-binding site confers trans-regulation by specific flavonoid-related R2R3 MYBs in diverse species. *Front. Plant Sci*. **8**, 1864 (2017). 10.3389/fpls.2017.01864 PMC5671642 29163590

[39] Comeskey DJ, Montefiori M, Edwards PJ, McGhie TK (2009). Isolation and structural identification of the anthocyanin components of red kiwifruit. J. Agric. Food Chem..

[40] Pilkington SM (2018). A manually annotated *Actinidia chinensis* var. chinensis (kiwifruit) genome highlights the challenges associated with draft genomes and gene prediction in plants. BMC Genomics.

[41] Werck-Reichhart D, Feyereisen R (2000). Cytochromes P450: a success story. Genome Biol..

[42] Chapple C (1998). Molecular-genetic analysis of plant cytochrome P450-dependent monooxygenases. Annu. Rev. Plant. Biol..

[43] Li W (2015). Gene expression profiling of development and anthocyanin accumulation in kiwifruit (*Actinidia chinensis*) based on transcriptome sequencing. PLoS ONE.

[44] Austin MB, Noel JP (2003). The chalcone synthase superfamily of type III polyketide synthases. Nat. Prod. Rep..

[45] Falcone Ferreyra ML, Rius S, Casati P (2012). Flavonoids: biosynthesis, biological functions, and biotechnological applications. Front. Plant Sci..

[46] Li Y., et al. A key structural gene, AaLDOX, is involved in anthocyanin biosynthesis in all red-fleshed kiwifruit (Actinidia arguta) based on transcriptome analysis. *Gene* **648**, 31–41(2018). 10.1016/j.gene.2018.01.022 29309888

[47] Pointer MR (1981). A comparison of the CIE 1976 colour spaces. Color Res. Appl..

[48] Nieuwenhuizen N. J., et al. Natural variation in monoterpene synthesis in kiwifruit: transcriptional regulation of terpene synthases by NAC and EIN3-like transcription factors. *Plant Physiol*. **167**, 124–58 (2015). 10.1104/pp.114.254367 PMC4378164 25649633

[49] Pilkington SM, Montefiori M, Jameson PE, Allan AC (2012). The control of chlorophyll levels in maturing kiwifruit. Planta.

[50] Hellens RP (2005). Transient expression vectors for functional genomics, quantification of promoter activity and RNA silencing in plants. Plant Methods.

[51] Wang T, Atkinson R, Janssen B (2007). Choice of Agrobacterium strain for transformation of kiwifruit. Acta Hortic..

