# Supplementary material for: Differential regulation of the anthocyanin profile in purple kiwifruit (Actinidia species)
Source: Hortic Res. 2019 Jan 1;6:3. doi: 10.1038/s41438-018-0076-4 (PMC6312553; doi:10.1038/s41438-018-0076-4)
Supplement: Supplementary file 1 — Supplementary Figures and Tables [file 41438_2018_76_MOESM1_ESM.docx]

**
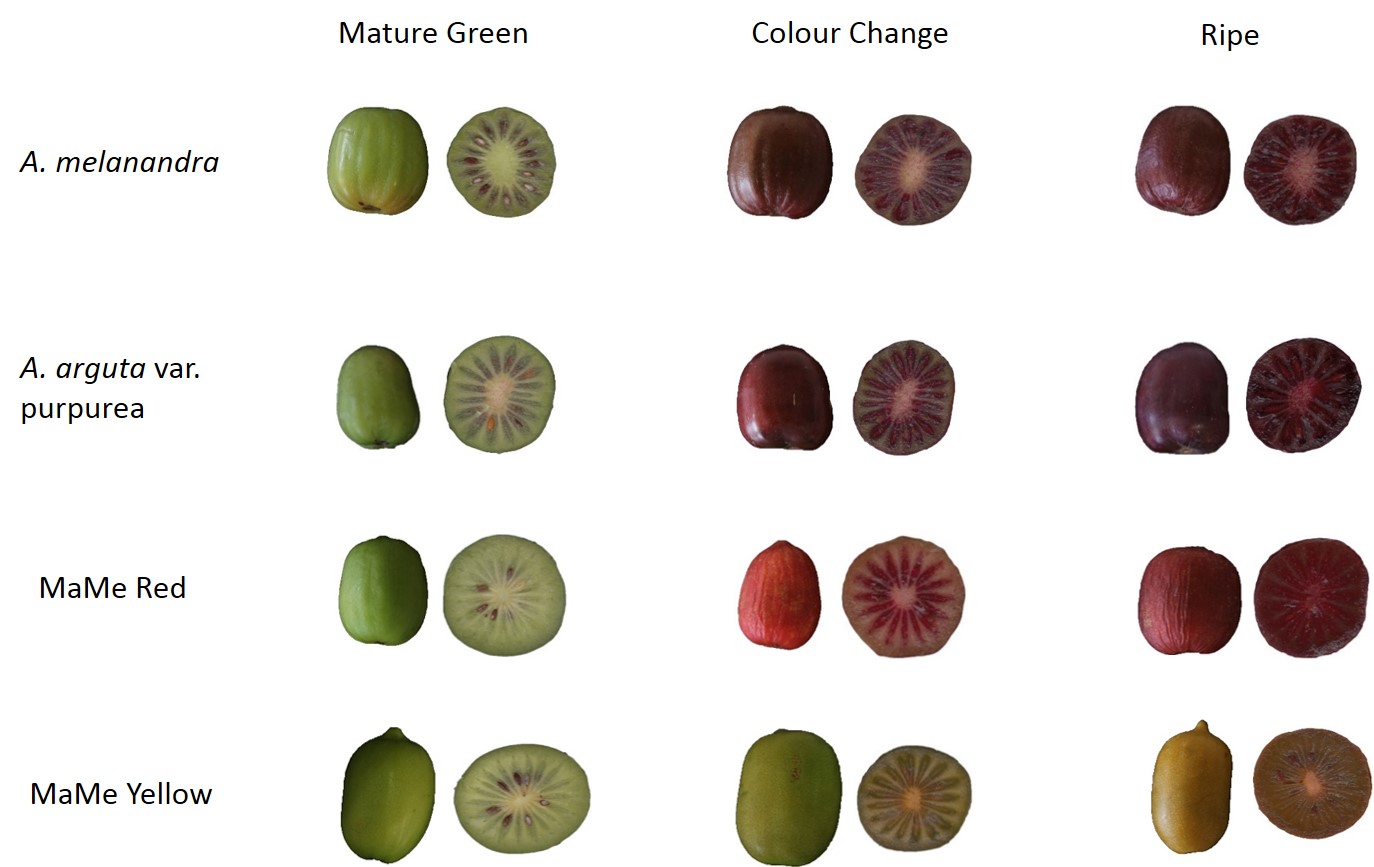
**

**Supplemental Figure 1:** Flat lay digital photographs of *A. melanandra, A. arguta* var *purpurea, A. macrosperma* x *A. melanandra* (MaMe) red and MaMe yellow showing the colour development on skin and flesh during mature green stage, colour change stage and ripe stage.

**
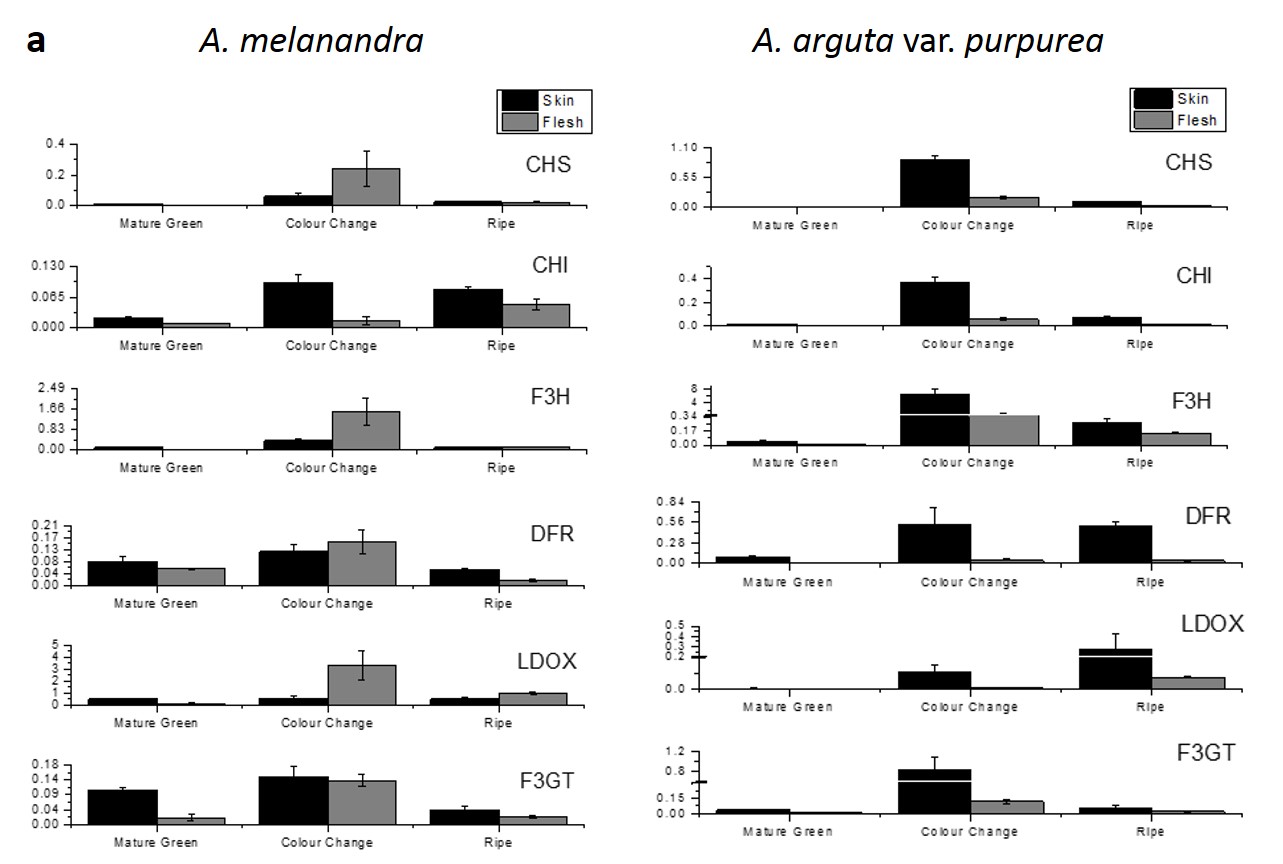

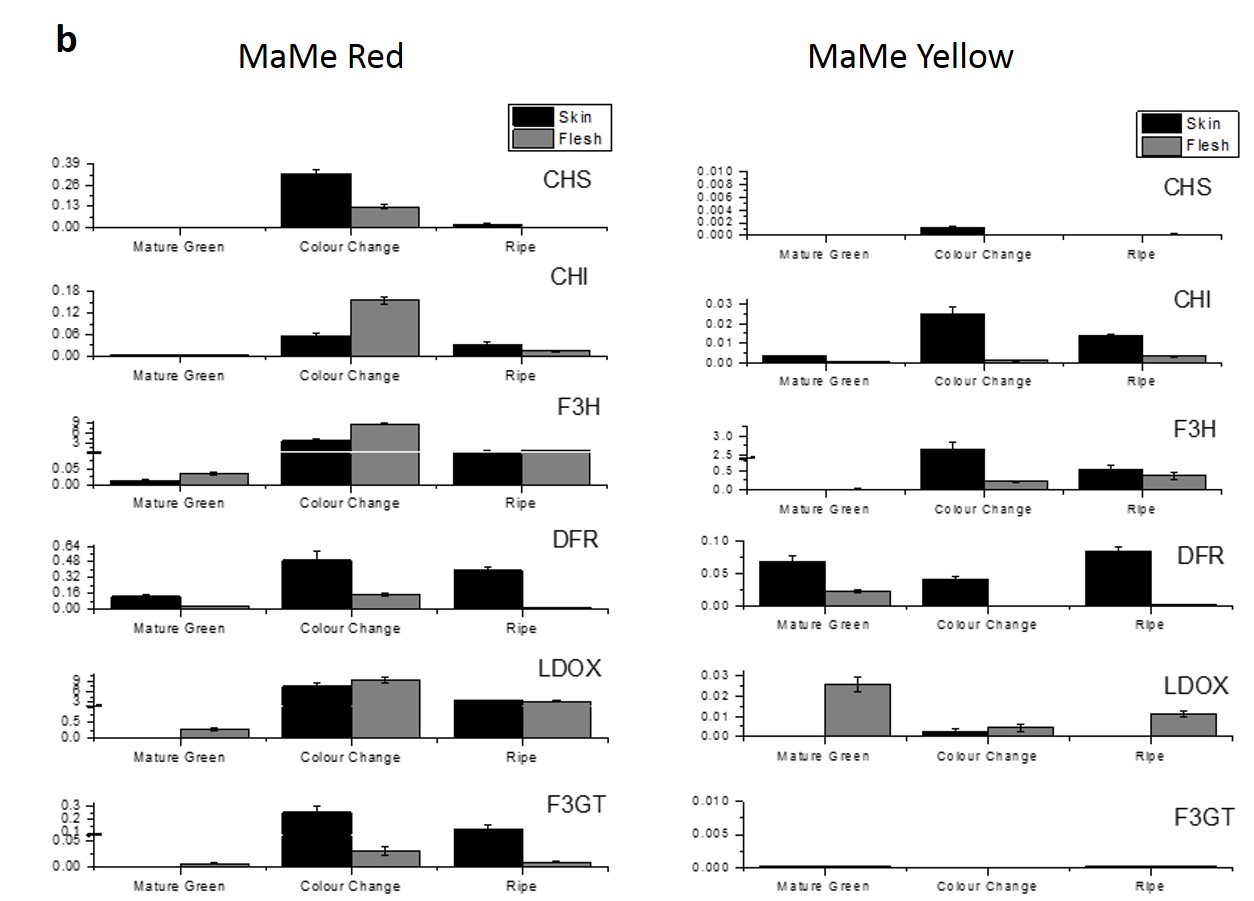
**

**Supplemental Figure 2:** Transcript expression analysis of anthocyanin biosynthetic genes in the skin and flesh tissue across the three developmental stages: mature green, colour change, and ripe. (a) Expression of genes in *A. melanandra* and *A. arguta* var. purpurea. (b) Expression of genes in two *A. macrosperma* x *A. melanandra* (MaMe) lines, red and yellow. CHS: chalcone synthase, CHI: chalcone isomerase, F3H: flavonone 3-hydroxylase, DFR: dihydroflavonol 4-reductase, LDOX: leucoanthocyanin dioxygenase, F3GT: flavonoid 3-*O-*glucosyltransferase. Error bars are SEM for 3 biological replicates.

**
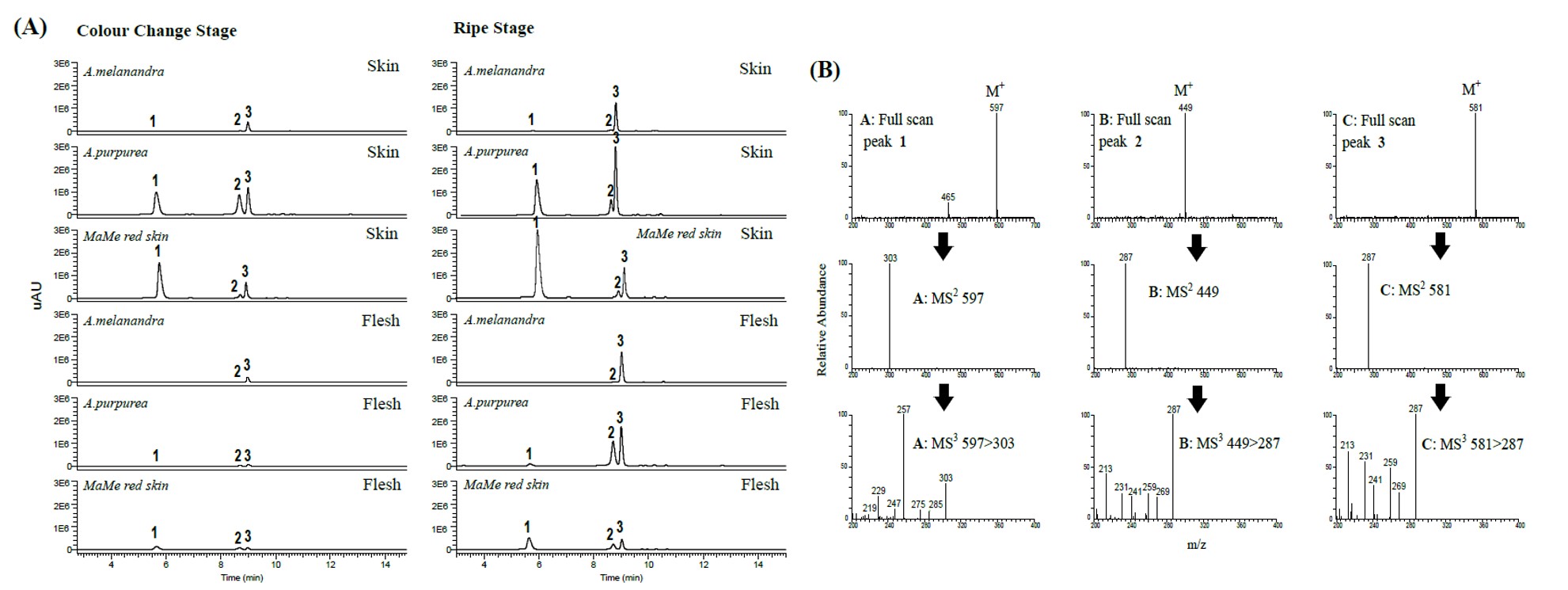
**

**Supplemental Figure 3:** LC-MS analysis of the cyanidin and delphinidin-based anthocyanins in the skin and flesh of *A. melanandra, A. arguta var purpurea* and MaMe red during colour change and ripe developmental stages. **(A)** PDA plots (520nm) of the skin and flesh of the ripening fruit lines showing detected anthocyanins; **1**, delphinidin; delphinidin 3-*O*-[2-*O*-(*β*-xylosyl)-*β*-galactoside] (dp-xylgal); **2**, cyanidin ; Cyanidin 3-*O*-*β*-galactoside (cy-gal); and **3**, cyanidin; cyanidin 3-*O*-[2-*O*-(*β*-xylosyl)-*β*-galactoside] (cy-xylgal). **(B)** MS spectra; A, fullscan, MS^2^ and MS^3^ data for peak **1**, dp-xylgal; B, fullscan, MS^2^ and MS^3^; data for peak **2**, cy-gal; and C, fullscan, MS^2^ and MS^3^ data for peak **3**, cy-xylgal.

**
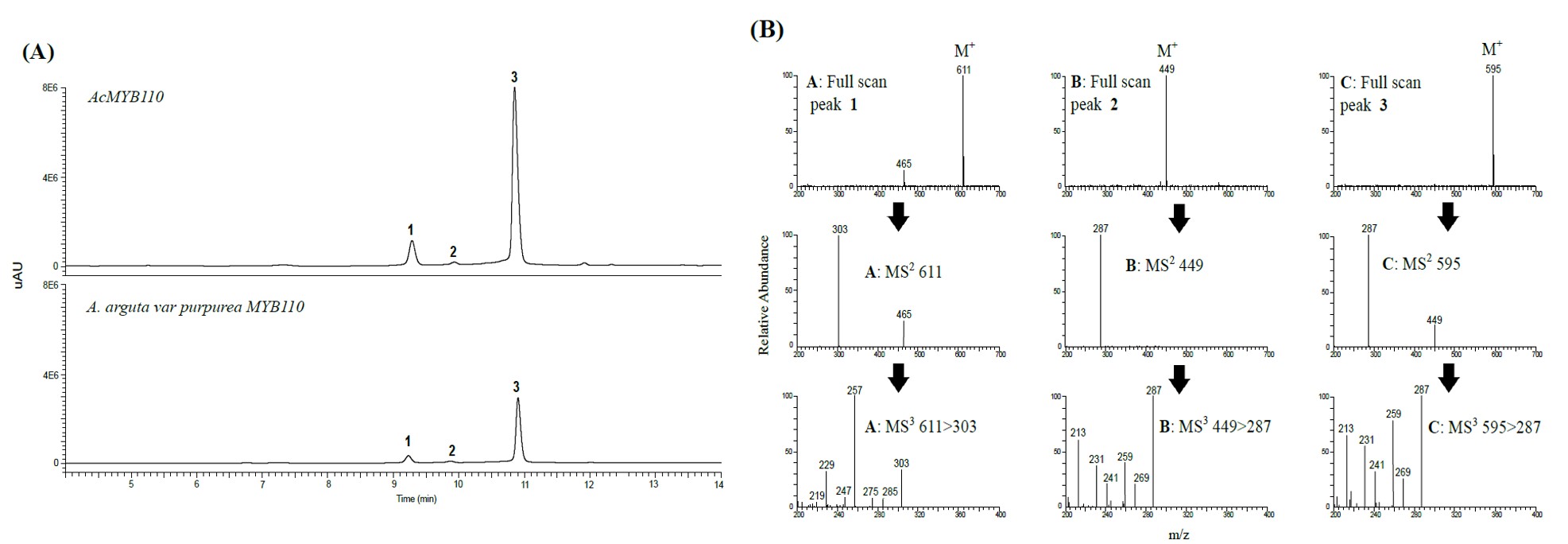
**

**Supplemental Figure 4:** LC-MS analysis of the cyanidin and delphinidin-based anthocyanins detected in *N. tabacum* leaf after transient expression of *AcMYB110* and *A. arguta* var *purpurea* *MYB110*. **(A)** PDA plots (520nm) showing detected anthocyanins; **1**, delphinidin 3-*O*-rutinoside (dp-rut); **2**, cyanidin 3-*O*-*β*-glucoside (cy-glu); and **3**, cyanidin 3-*O*xrutinoside (cy-rut). **(B)** MS spectra; A, fullscan,, MS^2^ and MS^3^ data for peak **1,** dp-rut; B, fullscanh MS^2^ and MS^3^ data for peak **2**, cy-glu; and C, fullscan, MS^2^ and MS^3^ data for peak **3**, cy-rut.

**Supplemental Table 1:** Quantification of anthocyanin in the skin and flesh tissue of *A. melanandra, A. arguta* var *purpurea, A. macrosperma* x *A. melanandra* (MaMe) red and MaMe yellow from mature green stage, colour change stage, and ripe stage. Anthocyanin were quantified as cyanidin-3-glucoside equivalents (µg/g FW) by HPLC.

|  | Mature Green Stage | Colour Change Stage | | | | Ripe Stage | | |  |
| --- | --- | --- | --- | --- | --- | --- | --- | --- | --- |
|  | Anthocyanin (µg/g) | Cyanidin-based anthocyanin (µg/g) | delphinidin-based anthocyanin (µg/g) | Total anthocyanin (µg/g) | Cyanidin-based anthocyanin (µg/g) | | delphinidin-based anthocyanin (µg/g) | Total anthocyanin (µg/g) | |
| *A. melanandra* skin | 0 | 125.8 | 0.0 | 125.8 | 148.8 | | 0.9 | 149.7 | |
| *A. melanandra* flesh | 0 | 84.1 | 0.4 | 86.2 | 157.8 | | 0.6 | 158.5 | |
| *A. purpurea* skin | 0 | 205.8 | 115.0 | 320.8 | 629.0 | | 346.8 | 975.8 | |
| *A. purpurea* flesh | 0 | 54.4 | 1.2 | 55.6 | 241.6 | | 8.4 | 250.0 | |
| MaMe red skin | 0 | 42.9 | 110.2 | 153.1 | 129.5 | | 348.6 | 478.1 | |
| MaMe red flesh | 0 | 36.3 | 31.1 | 67.5 | 43.3 | | 37.3 | 80.6 | |
| MaMe yellow skin | 0 | 0.0 | 0.0 | 0.0 | 0.0 | | 0.0 | 0.0 | |
| MaMe yellow flesh | 0 | 0.0 | 0.0 | 0.0 | 0.0 | | 0.0 | 0.0 | |

**Supplemental Table 2:** Pearson’s correlation analysis of the relationship between anthocyanin accumulation and expression of transcription factor at ripe stage. Significance level: p <0.05 = *; p <0.01 = **; p <0.001 = ***.

|  | Cyanidin-based anthocyanin | | Delphinidin-based anthocyanin | | Total anthocyanin accumulation | |
| --- | --- | --- | --- | --- | --- | --- |
|  | Pearson's coefficient | p-value | Pearson's coefficient | p-value | Pearson's coefficient | p-value |
| *MYB110* | 0.8456 | 0.0041** | 0.8724 | 0.0024** | 0.9482 | <0.001*** |
| *bHLH5* | 0.0811 | 0.4243 | 0.4362 | 0.14 | 0.2609 | 0.2662 |

**Supplemental Table 3:** Quantification of anthocyanin in the leaves of lines of *A. chinensis* over-expressing 35S:AcMYB10 and 35S:AcMYB110. Anthocyanin were quantified as cyanidin-3-glucoside equivalents (µg/g FW) by HPLC.

|  | Cyanidin (µg/g FW) | Delphinidin (µg/g FW) | Total anthocyanin (µg/g FW) |
| --- | --- | --- | --- |
| *A. chinensis* young leave | 8 | 0 | 8 |
| *A. chinensis* expanded leave | 0 | 0 | 0 |
| *A. chinensis* mature leave | 0 | 0 | 0 |
| 35S:MYB10 young leave | 33 | 1.7 | 34.7 |
| 35S:MYB10 expanded leave | 17 | 1.1 | 18.1 |
| 35S:MYB10 mature leave | 76 | 2.5 | 78.5 |
| 35S:MYB110 young leave | 643 | 26.95 | 669.95 |
| 35S:MYB110 expanded leave | 771 | 32.95 | 803.95 |
| 35S:MYB110 mature leave | 1105 | 25.05 | 1130.05 |

**Supplemental Table 4:** GenBank accession number of the genes used in this study.

| *A. chinensis* genes | Genome annotation number (Pilkington et al., 2018) | GenBank accession number (if available) |
| --- | --- | --- |
| ***CHS*** | Acc00260.1 | FG511211.1 |
| ***CHI*** | Acc03848.1 | FG404147 |
| ***F3H*** | Acc11906.1 | FG522061.1 |
| ***F3’H1*** | Acc18331.1 | FG420724 |
| ***F3’H2*** | Acc12813.1 | GU079684 |
| ***F3’5’H*** | Acc32390.1 | N/A |
| ***DFR1*** | Acc01005.1 | KF157393.1 |
| ***LDOX4*** | Acc28876.1 | KF157392.1 |
| ***F3GT*** | Acc20131.1 | GU079683.1 |
| ***MYB10*** | Acc00493.1 | N/A |
| ***MYB110*** | Acc10232.1 | KF311107.1 |
| ***bHLH5*** | Acc19563.1 | KY623715 |

**Supplemental Table 5:** Sequences of the primers used in this study.

| Gene (for qPCR) | Forward Primer (5’ – 3’) | Reverse Primer (5’ – 3’) |
| --- | --- | --- |
| ***CHS*** | ACAGCTTGACCACCTAAATGGGCTTA | CAAAGTGGAATAAAGCATGGCCAAT |
| ***CHI*** | GGAGGAGTTGACGGAATCCGTTG | AACTTTTCGACGGCTTTGGCCT |
| ***F3H*** | TGTACCCGCTCGCGATCCAG | ATTGCGTCTCTTTCGCAAGTTTCTTG |
| ***F3’H1*** | GGTGGCGTATGCTCCGGAAGA | GTGGCCCGCACTCACCAGTG |
| ***F3’H2*** | CGAAACGCCTCGAATCGTACG | CTGATCACGTTCAGGCTCACCG |
| ***F3’5’H*** | GATTTTGTGCCGTCGATTGCGT | AAGCTTGGGGCCATTAGGGTTTT |
| ***DFR1*** | GTCGGAGAAGCTGATTGGGATGGG | TGGCATTTCCATTGGGAGGGGTT |
| ***LDOX4*** | GTTCAGGAAGACCCAGGAGCTCG | TCCCCAAGACCTCAGAAGTCAAAAC |
| ***F3GT*** | TAGCCAAGCAGAGATCCGCTTCC | CAAGAATCCTTCTGGTAAGTACTGTTTCGA |
| ***EF1a*** | GCACTGTCATTGATGCTCCT | CCAGCTTCAAAACCACCAGT |
| ***MYB10*** | GACTTCCAGGAAGAACATCGAACGG | GGCGGGGACTTGGGGTGGGT |
| ***MYB110*** | ATATCTACAAGAGAAGAGCCGATACCCAA | TGGTTGAATGCTATCTGCAATGACAGT |
| ***bHLH5*** | AAGGGGAAGGCGGTGGATTCCG | GCACTGCAGCTCAACCAACG |
| ***AcMYB10*** | CTTCCGGGAAGAACATCAAACGA | GGCGGTGACTTGGGGTGG |
| ***AcMYB110*** | ATATCTACAAGAGAAGAGCCGATACCCAA | TGGTTGAATGCTATCTGCAATGACAGT |
| **Gene (For cloning)** | **Forward Primer (5’ – 3’)** | **Reverse Primer (5’ – 3’)** |
| ***F3’5’H promoter*** | TAATGCAATAAGAAGCACAGCG | GTTTGTGTAGTGTATTGCTTTGTG |
| ***F3’H promoter*** | ACTTCCTCATTGCACTTGCTC | TGTCTGTATAGTGGTGGTGGTG |
| ***F3’H1*** | ATGACTTCTCTGGCTCTTATTTT | TTACAAGCCATAGCCCATGC |
| ***F3’H2*** | ATGGAAACCCCTTCTTGGG | TCACAGCGCGTATAAATGAG |
| ***F3’5’H*** | ATGGCCATAGACATAATGTGGTTCA | TCACGAAACATAAGCACTTGGAGAC |
| ***MYB10*** | TAATAATCACTTGGTGTAGTGTAGG | GCAGACTCAACTCCATCTAATATTT |
| ***MYB110*** | CTTCAACAAATTGTTGTGATTTAGA | GGCCTCCGTCAAATATTTATTTTAG |
